# Inter-Individual Nectar Chemistry Changes of Field Scabious, *Knautia arvensis*

**DOI:** 10.3390/insects11020075

**Published:** 2020-01-22

**Authors:** Christine Venjakob, Sara Leonhardt, Alexandra-Maria Klein

**Affiliations:** 1 Ecosystem Functions, Institute of Ecology, Leuphana University of Lüneburg, Lüneburg, Universitätsallee 1, 21335 Lüneburg, Lower Saxony, Germany; 2 Agroecology, Department of Crop Sciences, University of Göttingen, Grisebachstrasse 6, 37077 Göttingen, Lower Saxony, Germany; 3 Biocenter—Am Hubland, Department of Animal Ecology and Tropical Biology, University of Würzburg, 97070 Würzburg, Bavaria, Germany; 4 Chair of Nature Conservation and Landscape Ecology, Faculty of Environment and Natural Resources, University of Freiburg, Tennenbacher Str. 4, 79106 Freiburg, Baden-Württemberg, Germany

**Keywords:** amino acids, carbohydrates, flower-visiting insects, insect nutrition, Jena Experiment

## Abstract

Nectar is crucial to maintain plant-pollinator mutualism. Nectar quality (nutritional composition) can vary strongly between individuals of the same plant species. The factors driving such inter-individual variation have however not been investigated closer. We investigated nectar quality of field scabious, *Knautia arvensis* in different grassland plant communities varying in species composition and richness to assess whether nectar quality can be affected by the surrounding plant community. We analyzed (with high performance liquid chromatography) the content of carbohydrates, overall amino acids, and essential amino acids. Amino acid and carbohydrate concentrations and proportions varied among plant individuals and with the surrounding plant community but were not related to the surrounding plant species richness. Total and individual carbohydrate concentrations were lowest, while proportions of the essential amino acids, valine, isoleucine, leucine (all phagostimulatory), and lysine were highest in plant species communities of the highest diversity. Our results show that *K. arvensis* nectar chemistry varies with the composition of the surrounding plant community, which may alter the taste and nutritional value and thus affect the plant’s visitor spectrum and visitation rate. However, the strong inter-individual variation in nectar quality requires additional studies (e.g., in semi-field studies) to disentangle different biotic and abiotic factors contributing to inter-individual nectar chemistry in a plant-community context.

## 1. Introduction

Plants are important bottom up partners of multitrophic interactions [1,2,3]. Interactions between plants and animals further drive important ecosystem functions and processes, such as herbivory, pollination or seed dispersal [1,4,5,6], because plants provide a habitat and resources for many animals [7,8]. These interactions in turn depend on the composition and diversity of the surrounding plant community [1]. Plant community composition directly determines the spectrum of interaction partners, as different animals require different spectra of plant species for resource acquisition [9] (pp. 190–200), while plant species diversity typically determines the diversity of higher trophic levels [10,11,12].

In general, resources essentially link plants and their interaction partners. For example, flower resources such as pollen and nectar, attract flower-visiting and pollinating insects, as they provide essential nutrients and thus represent the currency for their pollination success. Resource quality (i.e., the chemical nutritional composition of resources, such as nectar, pollen or leaves) varies between different plant species [13,14,15], and even between individuals of the same species and flowers of the same individual [16,17]. Resource quality can also change in relation to specific plant—insect interactions, likely to adjust to a specific interaction partner (e.g., specific flower visitor or herbivore species), as shown for vegetative tissue (e.g., leaves) [18,19,20,21,22]. The factors driving such inter-individual variation in resource quality are however still unclear, particularly for floral resources.

In plant-pollinator interactions, nectar plays a pivotal role; unlike pollen, it is consumed by most, if not all, flower visitors. Thus, pollinators visit flowers more frequently to collect nectar than pollen [23,24]. Nectar is produced in floral nectaries [25], but not in high volumes as it is costly for plants [26]. Nectar predominantly contains carbohydrates, namely two hexose monosaccharides (fructose and glucose) and a disaccharide (sucrose) [27,28] (pp. 215–264). Other components, such as amino acids, lipids [29], antioxidants, and alkaloids, are also present, albeit in much lower quantities, and can play an important nutritional (e.g., amino acids) role for pollinators [28,30,31]. After carbohydrates, amino acids are the most abundant nectar nutrient and encompass a wide range of different essential and non-essential amino acids sensu De Groot [32], which may vary in composition and concentration depending on the plant species [13]. Nectar amino acid and carbohydrate content can differ even between different cultivars (e.g., in rapeseed) [33]. Ratios of different macro-nutrient groups (e.g., of amino acids to carbohydrates) may also differ for floral nectar, but have been little investigated [30]. This is surprising, as nutrient ratios can be more important than overall content of different nutrients in determining nutritional quality for consumers in general [34,35] and flower visitors in particular [36,37,38,39].

Given the large inter- and intraspecific variation in nectar nutritional composition (henceforth termed nectar quality), differences in nectar quality likely contribute to pollinator community partitioning, as different flower visitors differ in taste preferences [14] and their nutritional requirements [18,37]. In fact, Garratt et al. [40] found that different apple varieties were pollinated by different pollinator communities likely due to variety-specific differences in nectar quantity and quality. Again, the factors underlying such inter-individual variation in nectar quality remain to be determined. They may comprise both biotic factors (e.g., community composition, species interactions) and abiotic factors (e.g., soil composition or pH).

Here, we investigate whether inter-individual variation in nectar quality (and thus potentially nectar attractiveness) varies with the surrounding plant community by investigating nectar quality of a common grassland (Arrhenatherion) plant species, the field scabious, *Knautia arvensis* (L.) Coult. Dipsacaceae, which typically attracts many flower-visiting insects [41] (pp. 557–562) or [42,43]. The study species was sown in 2002 in various plant communities differing in species richness and community composition (more details on the Jena Experiment [44]). We analyzed the composition of amino acids and carbohydrates as well as the ratios of carbohydrates to amino acids in nectar of *K. arvensis*, to relate nectar quality to changes in plant species richness and thus community composition.

We hypothesized that concentrations of carbohydrates, overall amino acids (AA), and essential amino acids (EAA) in nectar of *K. arvensis* will increase with increasing plant species richness, while their proportions should remain constant, because plants may be competing more strongly for pollinators in communities with more plant species, and thus pollinators present [11,45] compared to communities with less plant species. At least in some plant species, nectar composition can be phenotypically plastic and thus change following exposure to pollinators, as shown in *Helleborus foetidus* [46]. Such phenotypic plasticity in nectar chemistry could enable plants to adjust their nectar composition in response to an increased pollinator visitation frequency as likely found in species-rich plant communities. With regard to carbohydrate to amino acid ratios (henceforth referred to as C:AA and C:EAA ratios), we expected them to be constant across different communities, because different plant species typically have species-specific ratios of carbohydrates to amino acids [47].

## 2. Materials and Methods

### 2.1. Experimental Field Site

We collected nectar from *K. arvensis* grown in 7 different plots (i.e., from six 6 × 5.5 m + 3 × 3.5 m = 43.5 m^2^ community plots and one 1 × 1 m monoculture), in August 2010 (three out of seven plots) and June 2012 (all seven plots) (Figure S1 and Table S1). The community plots had different stabilized plant communities and were part of the Jena Experiment [44], which is a grassland biodiversity experiment located in Thuringia, Germany (50°55′ N, 11°35′ E; 130 meters above sea level (m a.s.l.)). Started in 2002, it comprises 82 plots, which were sown with 1, 2, 4, 8, 16 or 60 plant species from a 60-plant species pool, common in Central European mesophilic Arrhenatherion grasslands [44]. Specifically, 80 plots of 43.5 m^2^ (hereafter large plots) are located within 20 × 20 m squares comprising all diversity mixtures [48]. Monocultures of all species are either grown in large or small (1 × 1 m) plots (i.e., one monoculture per species) [48]. In June and September, all plots were mown, simulating traditional extensive hay meadow management. Plots were weeded three times during the year and all non-target plant species were removed. More detailed description of the experiment is given in a study by Roscher et al. [44].

We collected *K. arvensis* nectar from all plots comprising sufficient (i.e., a minimum of five plants) *K. arvensis* individuals, namely from one small monoculture, one 4-species, three 8-species, and two 16-species plots (see Figure S1 for plot distribution and distances between plots). As plot size of the monoculture (8 replicates) was smaller than of the mixtures (47 replicates), we tested if plot size affected any of our nectar response variables. As plot size did not explain the nectar variables, we did not consider it in our analyses described below.

All plant species, including *K. arvensis*, were sown in 2002 and since then maintained by regular mowing and weeding of non-target plant species three times per year. At plots with plant species mixtures, other plant species typically flowered simultaneously and thus likely competed for pollinators with *K. arvensis* (see Table S1 for a detailed list of plant species flowering at each plot). We considered one plant individual as one sample and one inflorescence typically consists of approximately 55–100 flowers [49], which are arranged in a dense flower head and mostly provide enough nectar for one sample (>1 µL). We took great care not to scratch flowers or inner tissue or to contaminate samples with pollen [50] during nectar sampling. However, even if slight contamination with pollen grains occurred, it unlikely affected the amino acid composition of nectar as shown for *Aloe marlothii* nectar [51] (p. 206). We consequently collected a minimum of five samples per plot (7.86 ± 4.02).

### 2.2. Nectar Sampling

For standardized nectar sampling, nectar was collected from at least five *K. arvensis* individuals per plot between 10 a.m. and 2 p.m. on sunny or light cloudy days. Five was the minimum number of samples that we aimed for. Where possible, we collected more samples to obtain a more robust dataset. The day before sampling, we placed gauze bags (mesh size 0.8–1.00 mm) around at least five inflorescences (8.14 ± 3.93) to prevent early foraging pollinators from depleting nectar standing crop [52]. Since nectar secretion is typically dynamic and highly variable over the course of one day, we rapidly assessed nectar production, prior to the actual sampling, through repeated sampling and measuring at the site using a hand-held refractometer. We found *Knautia arvensis* to produce nectar all day long. Nectar was collected from several florets using microcapillaries with a minimum volume of 1 µL (pipetting aid and a disposable capillary; minicaps^®^, Hirschmann Laborgeräte GmbH & Co. KG, Eberstadt, Germany) [53]. We chose and sampled florets of all ages to average across age-specific differences for each sampled plant individual. Nectar samples of each plant individual were stored in clean and autoclaved 1.5 mL Eppendorf tubes (Safe-Lock Tubes, Eppendorf AG, Hamburg, Germany) and kept in a cool box in the field before freezing at −20 °C.

### 2.3. Sample Preparation

To analyze the amino acid and carbohydrate composition in nectar, samples were re-dissolved in 100 µL of 99.8% ethanol (CHROMASOLV^®^, Sigma-Aldrich Laborchemikalien GmbH, Hannover, Germany), centrifuged for 5 min (158 g, Mikro 22 R, Hettich Lab Technology, Schwerin, Germany), and transferred from capillaries into Eppendorf tubes using a pipetting aid. Samples were kept in a DURAN^®^-desiccator (CARL ROTH GMBH + CO. KG, Karlsruhe, Germany) to completely evaporate alcohol at 20 °C, before adding 50 µL ultra-pure water (Siemens AG, Barsbüttel, Germany) for centrifuging (3 min) to remove potential left-over precipitates. From the supernatant, 48 µL were pipetted into 1 mL glass vials for HPLC analytics (Agilent Technologies, Böblingen, Germany), equipped with 250 μL pulled-point glass inserts (Agilent Technologies, Böblingen, Germany), and frozen at −20 °C prior to chemical analyses. Before analysis, we waited until the samples adjusted to the ambient temperature and ensured that no precipitation occurred after taking the prepared samples out of the freezer.

### 2.4. Amino Acid and Carbohydrate Analysis

Amino acids and carbohydrates were analyzed using high performance liquid chromatography (HPLC) from Agilent Technologies 1260 Series provided with an Agilent 1260 Infinity Quaternary Pump (G1311C, Agilent Technologies, Böblingen, Germany), an Agilent 1260 Infinity Standard Autosampler (G1329B), and an Agilent 1260 Infinity Thermostatted Column Compartment (G1316A), to maintain the temperature for amino acids at 40 °C and for carbohydrates at 30 °C. 

Amino acids were separated on a Zorbax Extend-C18 column (3.0 × 150 mm, 3.5 µm, Agilent Technologies, Böblingen, Germany), preceded by a guard column Zorbax Extend-C18 (2.1 × 12.5 mm, 5 µm, Agilent Technologies, Böblingen, Germany), and were detected by an Agilent 1260 Infinity System Diode Array Detector (DAD, G4212B) with a flow rate of 1 mL min^−1^. Prior to injection, amino acids were derivatized with either ortho-phthalaldehyde (OPA, Agilent Technologies, Böblingen, Germany, for primary amino acids: alanine, arginine, aspartic acid, cystine, glutamic acid, glycine, histidine, isoleucine, leucine, lysine, methionine, phenylalanine, serine, threonine, tyrosine, and valine) or 9-fluorenylmethyl chloroformate (FMOC, Agilent Technologies, Böblingen, Germany, for proline) [54,55,56]. Amino acids were separated by a solvent gradient with a buffer (1 L ultra-pure water, 10 mM Na_2_HPO_4_, 10 mM Na_2_B_4_O_7_, 0.5 mM NaN_3_, pH 8.2) used as polar phase and acetonitrile-methanol-water (45%:45%:10% (*v/v*), all CHROMASOLV^®^, Sigma-Aldrich Chemie GmbH, Munich, Germany) used as non-polar phase [54,55]. We started with a 2%:98% non-polar to polar phase, then gradually changed the ratio to 57%:43% for 13 min, until finally increasing the non-polar phase to 100% for a period of 2 min, followed by a re-equilibration to 2%:98% non-polar to polar phase for about 9 min [54]. Solvent flow rate was 0.750 mL min^−1^ [54].

Carbohydrates were separated on a NH2 column (Zorbax: 4.6 × 250 mm, 5 µm, Agilent Technologies) preceded by a NH2 guard column (Zorbax: 4.6 × 12.5 mm, 5 µm, Agilent Technologies) under isocratic conditions using an elution buffer with 78%:22% (*v/v*) acetonitrile and ultra-pure water and a flow rate of 1.5 mL min^−1^. Carbohydrates were detected by a refractive index detector (RID, Agilent 1260 Infinity, G1362 A) [57].

Four different concentrations of a standard comprising 17 amino acids (Amino Acid Standard solution, Sigma-Aldrich Laborchemikalien GmbH, Hannover, Germany) or three carbohydrates (sucrose, fructose, and glucose, HPLC grade, Sigma-Aldrich Laborchemikalien GmbH, Hannover, Germany) were run every five samples as an external reference. All amino acids and carbohydrates in nectar samples were identified based on standard reference compounds. HPLC control and compound quantification was carried out with Agilent ChemStation for LC 3D systems (Agilent Technologies, Böblingen, Germany).

### 2.5. Observations of Flower Visitors

Flower-visiting insects, such as honeybees, bumblebees, solitary bees, and hoverflies, were surveyed within the framework of the Jena Experiment on a subset of plots between May and August 2011 (see [58] for details on how flower visitors were observed). Thus, nectar sampling and flower-visitor observations were performed at different years. We extracted all observations on flower visitors to *K. arvensis* for those plots for which we also had collected nectar (i.e., two 8-species plots and one 16-species plot). Flower visitors were grouped as honeybees, bumblebees, solitary bees, and hoverflies for subsequent analyses, and we defined all solitary bees as non-eusocial Apidae [59]. We finally summed all flower visitors across all observations performed in 2011 and calculated per plot the Shannon diversity index, total number of species, total number of individuals, and number of individuals for the different flower-visiting groups for each plant community.

### 2.6. Statistical Analyses

We investigated whether nectar from *K. arvensis* plants grown in plant communities that differed in species richness (i.e., *K. arvensis* monocultures and communities with 4, 8, and 16 plant species) and composition of plants differed in the composition of carbohydrates and/or amino acids as well as the ratio of all carbohydrates to all amino acids. We used permutation tests based on Bray–Curtis distances between substances (i.e., Adonis in the vegan R package) to test for an effect of community richness. Separate permutation tests were performed for concentrations (in mg/mL) and proportions of individual carbohydrates and amino acids. Proportions of individual compounds were obtained by dividing the concentration of each individual carbohydrate/amino acid by the total concentration of all carbohydrates/amino acids analyzed. When significant differences between communities were found, we subsequently analyzed differences between plant species in the concentrations and/or proportions of all individual carbohydrates/amino acids. We further assessed community-specific differences for total carbohydrate and amino acid concentrations, the concentration and proportion of essential amino acids (EAA), and non-essential amino acids (nEAA) and the C:AA, C:EAA, and C:nEAA ratios. Three outliers were excluded from the original dataset for the amino acid analyses.

Due to the nested plot design, from which the samples were taken, we always tested first whether the sample plot significantly affected the explained variance by composing both generalized linear models (GLMs) and generalized linear mixed effect models (GLMMs) with plant species richness level entered as a categorical fixed factor and the plot from which the sample was taken as a random factor. Models were compared using the Akaike information criterion (AIC) and likelihood ratio tests (Adonis command in the lme4 R package). AIC values were always similar for GLMMs and GLMs, which renders the application of permutation tests and GLMs (not accounting for random plot effects) for richness level-specific differences in compound compositions as valid. The lack of a plot effect further indicates that plot, and thus the corresponding plot-specific plant community composition, did not significantly explain the observed variation in nectar chemistry. Significant variation in nectar quality between communities as revealed by GLMs was subsequently analyzed with Tukey’s post hoc tests. Preliminary data screening revealed that total carbohydrate concentrations as well as concentrations of all individual carbohydrates (i.e., glucose, fructose, and sucrose) significantly increased over the course of the day (all *r* > 0.3, *p* < 0.03, Spearman correlation). We therefore included “time of day” as a random factor in all models including carbohydrate concentrations (using the lmer function in the lme4 R package). Due to unequal plot numbers between years and different sampling months (August in 2010 and June in 2012), we did not include year as random factor in the models. Moreover, differences between years were relatively low (i.e., total sugars: 61.4 ± 47.1 mg/mL in 2010 and 66.0 ± 95.8 mg/mL in 2012; total AA: 1.9 ± 1.6 mg/mL in 2010 and 2.3 ± 2.8 mg/mL in 2012), indicating that variation was better explained by factors other than the year.

Response variables were always tested for normality and homogeneity of variances using the Shapiro–Wilk normality test (stats R package, version 4.0.0) and graphical tools as suggested by [60]. We log or square root (concentrations, ratios) or arcsine square root (proportions) transformed the data when these requirements were not met. Due to multiple usage of the same dataset, we only considered *p*-values below 0.01 as significant.

We finally used the carbohydrate and amino acid proportions and concentrations of each plant sample to visually display differences between richness levels using non-metrical dimensional scaling (NMDS, R package vegan) also based on Bray–Curtis distances between substances. All analyses were performed in R, version 3.1.3 [61].

## 3. Results

**Figure 1:**
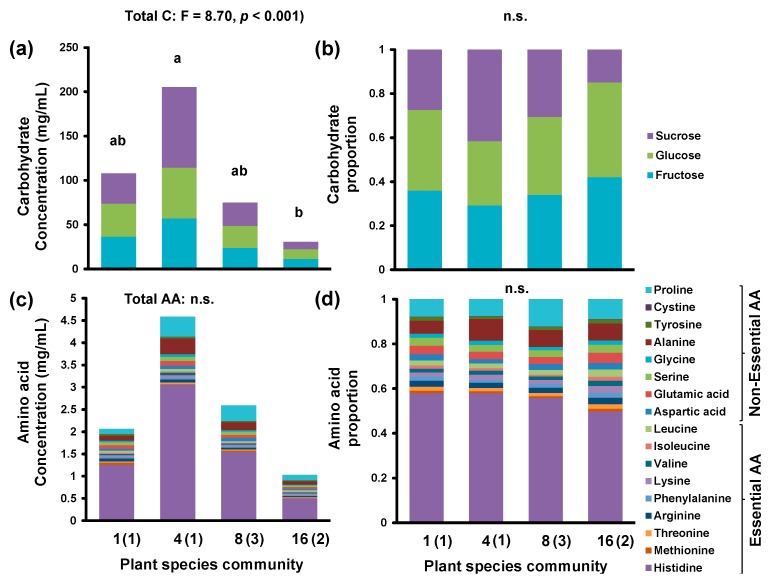
Carbohydrate (**a**) concentrations and (**b**) proportions as well as amino acid (**c**) concentrations and (**d**) proportions in nectar of *Knautia arvensis* growing in different plant communities with one, four, eight, and 16 different plant species. Concentration is given in mg/mL. Amino acids are divided into non-essential and essential amino acids (sensu [32]). Numbers in parentheses give the number of plots sampled per plant community. Letters above the bars indicate the significance of differences between plant species communities, while n.s. indicates no significance.

Overall, nectar amino acid and carbohydrate concentrations and proportions varied among *K. arvensis* individuals, but also with community composition and thus species richness (Table 1, Figure S2). Nectar generally contained similar concentrations/proportions of the three major carbohydrates (glucose, fructose, and sucrose), but different concentrations/proportions of amino acids (Table 1, Figure 1, Figure S2). Histidine was most prominent and accounted for more than 50% of all amino acids in most samples (see Table 1, Figure 1c,d, Figure S2), with proline and alanine representing the second most prominent amino acids (each present in more than 10 times lower concentrations than histidine; Table 1, Figure 1c,d, Figure S2).

**Figure 2:**
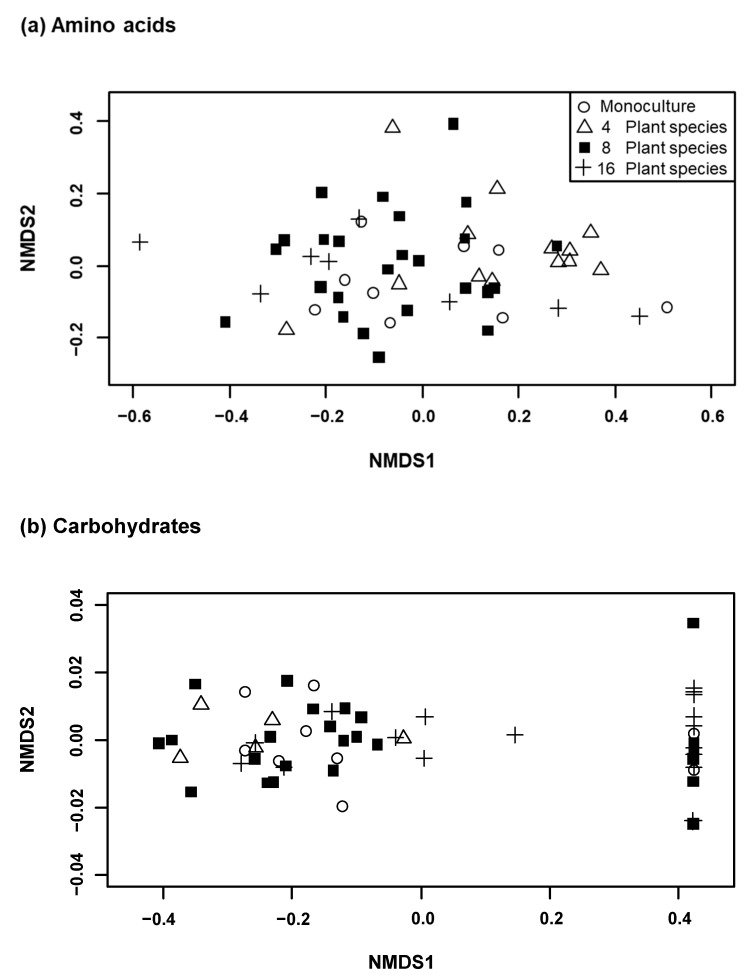
Non-metrical dimensional scaling (NMDS) based on (**a**) proportions of amino acids and (**b**) concentrations of carbohydrates in floral nectar of *Knautia arvensis*. Each symbol represents a sample of different plant communities: open circles = monoculture, open triangles = four plant species communities, filled black squares = eight plant species communities, and black crosses = 16 plant species communities. Note that all samples accumulating at the right side of the graph lack sucrose, likely because sucrose had already been hydrolyzed to fructose and glucose by yeast and/or bacteria which naturally occur in nectar [63].

*K. arvensis* individuals from communities differing in plant species richness had specific compositions of amino acids when proportions were considered (Adonis: *r^2^* = 0.12, *p* < 0.01; Table 1, Figure 2a, Figure S2) and of carbohydrates when concentrations were considered (*r^2^* = 0.29, *p* < 0.001; Table 1, Figure 2b, Figure S2). With regard to concentrations of amino acids their composition tended to show community-specific profiles (*r^2^* = 0.11, *p* = 0.02), as did proportions of carbohydrates (*r^2^* = 0.18, *p* = 0.02).

Concentrations of all amino acids tended to differ between communities (GLM: *F* = 2.56, *p* = 0.06) and were highest in the four-species community (Figure 1c, Table 1, Figure S2). Proportions of all essential amino acids did not differ between communities (*F* = 1.94, *p* = 0.13) (Table 1, Figure 1d, Figure S2), but the individual essential amino acids valine (*F* = 5.16, *p* < 0.01), isoleucine (*F* = 6.16, *p* < 0.001), leucine (*F* = 5.56, *p* < 0.01), and lysine (*F* = 4.71, *p* < 0.01) were all found in significantly higher proportions in the nectar of plants of the 16, rather than the eight, species community (Tukey’s test: all *p* < 0.01). There were no significant differences when comparing with the other plant species communities (monoculture and four plant species community) (Tukey’s test: all *p* > 0.01) (Table 1, Figure 1, Figure S2).

The only non-essential amino acids that tended to proportionally differ between communities was aspartic acid (GLM: *F* = 3.64, *p* = 0.02) with the highest proportions in the *K. arvensis* monoculture and the 16 plant community (Tukey’s test: *p* = 0.01; Table 1, Figure 1d, Figure S2).

With regard to carbohydrates, both total carbohydrate concentration (*χ^2^* = 24.84, *p* < 0.001) and the concentrations of individual carbohydrates (glucose: *χ^2^* = 23.58, *p* < 0.001; fructose: *χ^2^* = 23.07, *p* < 0.001; sucrose: *χ^2^* = 26.40, *p* < 0.001) were lowest in the 16 species community and highest in the four species community (Table 1, Figure 1a, Figure S2). Trends were the same for sucrose proportions (*F* = 3.40, *p* = 0.02), but reversed for proportions of glucose (*F* = 3.40, *p* = 0.02) and fructose (*F* = 3.40, *p* = 0.01) (Table 1, Figure 1b, Figure S2).

**Figure 3:**
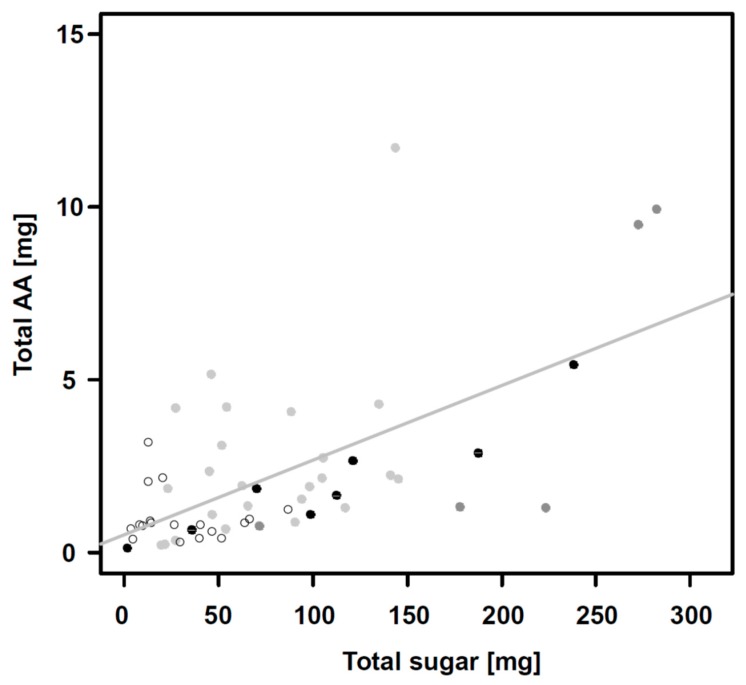
Ratio of total amino acids to total carbohydrates in mg (C:AA) in nectar of *Knautia arvensis* grown in different plant species richness communities. Each dot represents one sample with different colors indicating different plant species communities: black dots = monoculture, dark grey = 4, light grey = 8, white = 16 plant species community.

Nectar generally contained more carbohydrates than amino acids (Figure 1 and Figure 3), but the ratio of carbohydrates to amino acids (C:AA) varied strongly between individual plants (Table 1, Figure 3, Figure S2) and ranged from C:AA ratios of 4:1 (16 species community) to 170:1 (four species community) with a mean value (±standard deviation) of C:AA 49 (±37):1 (Figure 3). However, C:AA ratios did not show clear community-specific differences (*F* = 1.96, *p* = 0.13), and neither did C:EAA (*F* = 0.27, *p* = 0.85) and C:nEAA (*F* = 0.97, *p* = 0.41) ratios (Table 1, Figure 3, Figure S2).

Though the Shannon index of flower-visiting insects was slightly higher in one of the eight plant species communities than in the 16 plant species community (Table 2 and Table S2), the number of individuals of flower-visiting guilds increased with plant species community (from eight to 16 plant species community) as did the total numbers and species richness of flower visitors (Table 2, for more details Table S2). 

## 4. Discussion

Our results confirm strong inter-individual variation in nectar chemistry and further show that the nutritional composition of floral nectar of *K. arvensis* can vary strongly with the surrounding plant species community. For example, proportions of the essential amino acids, valine, isoleucine, leucine, and lysine and the non-essential amino acid, aspartic acid, differed between communities and were all highest in the 16 species community. However, contrary to our hypothesis, we found the observed variation in nutrient concentrations to be independent of the plant species richness in the surrounding plant community. Inter-individual variation in *K. arvensis* nectar chemistry therefore appeared to be affected by genetic differences between individuals, by abiotic factors or by the composition of the surrounding community rather than by its species richness. In fact, the spectrum of plant species co-occurring and in particular co-flowering with *K. arvensis* differed between plots (see Table S1). As the competitiveness of a specific plant species can differ with the surrounding plant community [63,64], it is possible that *K. arvensis* experienced different, and community-dependent, levels of competition at different plots, which may have indirectly affected its nectar chemistry. In interaction with subtle, potentially also plant-community mediated, differences in soil quality (i.e., concentration and composition of soil nutrients, microbial communities), such community-dependent competition may (at least partly) explain the considerable variation in nectar chemistry both within and between plant communities [58,65,66,67]. Community-dependent competition can also be caused by different intensities of wind-pollinated plant species [65]. It does, however, not explain the large variation in nectar chemistry observed for different individuals even within the same plot. Nutrient concentrations in nectar can vary due to water evaporation over the course of a day and in relation to ambient relative humidity [68], resulting in nectar viscosity increasing with increasing temperatures and/or decreasing humidity [53]. Although nectar sampling was confined to a period of 4 h (i.e., took place between 10 a.m. and 2 p.m.) in our study, the total carbohydrate concentration in nectar significantly increased over this period (Spearman rank correlation test: *r* = 0.33, *p* = 0.02) and ranged from mean 37.78 (± 20.01) mg/mL at 10 a.m. to mean 103.62 (± 92.60) mg/mL at 2 p.m. (data pooled for both years). This significant effect of sampling time indicates that abiotic factors can determine nectar chemistry more strongly than biotic factors, such as the surrounding plant community. However, sampling time did not affect total amino acid concentration (*r* = 0.03, *p* = 0.86). It therefore remains unclear which alternative factors caused the variation in nectar amino acid content (coefficient of variation (CV): 1.09) which was even slightly higher than nectar carbohydrate content (CV: 0.88). Additional variation may have been caused by differences in the biomass and/or density of *K. arvensis* plants between plots, differences in pollinator communities and thus visitation frequencies between plots [11,58], differences in plot sizes, and/or by flower handling and sample collection, although we took extreme caution to standardize sampling.

The observed variation in nectar chemistry may in turn have had strong effects on flower visitors. For example, honeybees typically prefer sugar solutions with essential amino acids over sugar solutions with non-essential amino acids [69], but can be deterred by specific amino acids (e.g., alanine [68] or glycine [67]), while other amino acids (e.g., isoleucine) appear to act as a feeding stimulant and increase nectar consumption [70]. Differences in the concentration or proportion of specific amino acids can consequently attract or deter specific flower visitors and differences in amino acid proportions and ratios may thus structure visitation patterns. In fact, the proportional increase in phagostimulatory attractive amino acids (i.e., essential amino acids and isoleucine) in *K. arvensis* nectar at 16 species plots may (among others) explain why most honeybees (*Apis mellifera* Linnaeus, 1758) were observed on *K. arvensis* plants in the 16 species plot (Table 2 and Table S2) [59,62].

The general prevalence of histidine in *K. arvensis* nectar (which could account for <50% of total amino acids) is intriguing and differs from other plant species where histidine proportions commonly lie between 12% and 16% (for different *Brassica napus* cultivars [33]) or below 3% (for *Maurandya barclayana*, *Lophospermum erubescens*, and *Brassica napus* [69]). Consumers of *K. arvensis* nectar will thus likely over-eat histidine if they aim to obtain sufficient amounts of the other amino acids (or a balanced C:AA ratio), with unknown consequences for their health or behavior. However, histidine may act as a repellent to honeybees as shown by Hendriksma et al. [67], where nectar with histidine was less frequently consumed than nectar with glycine and cysteine.

In contrast to proportions of specific amino acids, carbohydrate to amino acid ratios (C:AA, Table S2) showed, as expected, no community-specific pattern, but also varied strongly between plant individuals. The carbohydrate to amino acid ratio was generally carbohydrate biased, which agrees with nectar’s major role as a carbohydrate source [71] (pp. 142–159). It also meets the nutritional needs of most flower visitors, such as honeybee and bumblebee workers, which typically prioritize carbohydrate over (essential) amino acid intake, even over-eat amino acids to obtain sufficient carbohydrates, and perform generally better on carbohydrate-rich diets [36,72]. Carbohydrates are important for flight performance in flower visitors [73].

For future work, we propose to repeat similar investigations and analyses, ideally under more controlled conditions (e.g., in greenhouses) to reduce sources of variation. It would further be worthwhile expanding nectar sampling and pollinator observations to more plant communities and species to directly relate nectar quality, flower visitor spectra, visitation rates, and floral constancy of pollinators to the composition of the direct and wider surrounding community. This is essential for understanding which factors drive inter-individual variation in nectar quality and how interactions between resource (nutritional) characteristics and the environment structure flower visitor interactions.

## 5. Conclusions

Both carbohydrate and amino acid content in nectar varied between *K. arvensis* individuals as well as between the different plant species richness levels of plant communities. However, there were significant differences in proportions in some essential and phagostimulatory amino acids in nectar of *K. arvensis* plants in plant species-rich communities, while the inhibiting amino acid histidine tended to be less available. This suggests that *K. arvensis* nectar is more palatable to insects when plants grow in plant communities with high plant species richness. However, the strong inter-individual variation in nectar quality requires additional studies (e.g., in semi-field conditions).

## Figures and Tables

**Table 1:** Nectar volume, concentration (mg/mL), proportion, and ratios of chemical components, such as amino acids and carbohydrates shown here as mean per plant species level with standard deviation (SD).

Plant Species Community ^1^			1		4		8		16	
N Samples ^2^			8		5		24		18	
			Mean	±SD	Mean	±SD	Mean	±SD	Mean	±SD
*Volume Nectar ^3^*	µL		3	4.11	3.31	2.71	2.73	2.73	4.35	2.76
Amino Acids										
Asp ^4^	**C**	mg/mL	0.05	0.03	0.08	0.09	0.07	0.07	0.03	0.03
Glu	**O**		0.08	0.08	0.11	0.09	0.07	0.08	0.04	0.03
Ser	N		0.07	0.07	0.09	0.07	0.07	0.08	0.03	0.02
His	**C**		1.26	1	3.06	3.38	1.55	1.65	0.5	0.31
Gly	**E**		0.04	0.04	0.06	0.04	0.04	0.05	0.02	0.01
Thr	**N**		0.03	0.03	0.04	0.03	0.03	0.02	0.02	0.01
Arg	**T**		0.06	0.09	0.06	0.04	0.04	0.03	0.03	0.02
Ala	**R**		0.11	0.09	0.35	0.31	0.17	0.14	0.08	0.07
Tyr	**A**		0.04	0.04	0.04	0.02	0.03	0.03	0.02	0.02
Cystine	**T**		0	0	0.01	0.01	0	0	0	0
Val	**I**		0.03	0.03	0.05	0.04	0.03	0.02	0.02	0.02
Met	**O**		0.04	0.1	0.01	0.02	0.02	0.05	0.01	0.01
Phe	**N**		0.03	0.03	0.03	0.02	0.03	0.03	0.02	0.02
Ile			0.03	0.02	0.03	0.02	0.01	0.01	0.02	0.01
Leu			0.04	0.04	0.06	0.03	0.04	0.03	0.03	0.03
Lys			0.04	0.04	0.07	0.04	0.04	0.02	0.03	0.03
Pro			0.12	0.09	0.45	0.52	0.35	0.37	0.12	0.17
Total AA ^5^			2.07	1.66	4.59	4.71	2.59	2.4	1.03	0.74
EAA ^6^			1.57	1.31	3.41	3.59	1.79	1.75	0.69	0.43
nEAA ^7^			0.49	0.4	1.18	1.12	0.8	0.69	0.34	0.33
Asp ^4^	**P**		0.03	0.01	0.02	0.01	0.03	0.01	0.03	0.01
Glu	**R**		0.04	0.01	0.03	0.02	0.03	0.02	0.05	0.02
Ser	**O**		0.04	0.02	0.03	0.02	0.03	0.02	0.04	0.02
His	**P**		0.58	0.13	0.58	0.12	0.56	0.09	0.5	0.13
Gly	**O**		0.02	0.01	0.02	0.01	0.02	0.01	0.02	0.01
Thr	**R**		0.02	0.01	0.01	0.01	0.02	0.01	0.02	0.01
Arg	**T**		0.03	0.01	0.02	0.02	0.02	0.01	0.03	0.01
Ala	**I**		0.06	0.02	0.1	0.03	0.08	0.03	0.08	0.02
Tyr	**O**		0.02	0.01	0.01	0.01	0.01	0.01	0.02	0.01
Cystine	**N**		0	0	0	0	0	0	0	0
Val			0.02	0.01	0.02	0.01	0.02	0.01	0.02	0.01
Met			0.01	0.02	0.01	0.02	0.01	0.01	0.01	0.02
Phe			0.02	0.01	0.01	0.01	0.01	0.01	0.02	0.01
Ile			0.01	0.01	0.01	0.01	0.01	0.01	0.02	0.01
Leu			0.02	0.01	0.02	0.01	0.02	0.01	0.03	0.01
Lys			0.02	0.01	0.02	0.01	0.02	0.01	0.03	0.01
Pro			0.08	0.06	0.08	0.03	0.12	0.07	0.09	0.05
Total AA ^5^			1	-	1	-	1	-	1	-
EAA ^6^			0.73	0.09	0.71	0.04	0.68	0.07	0.69	0.08
nEAA^7^			0.27	0.09	0.29	0.04	0.32	0.07	0.31	0.08
**Carbohydrates**										
Fructose	**C**	mg/mL	36.44	25.51	57.25	22.67	23.95	12.76	11.21	7.39
Glucose	**O**		37.12	27.73	57.04	22.2	24.6	12.65	11.25	7.16
Sucrose	**N**		34.41	27.43	91.14	44.25	26.54	23.9	8.12	11.42
Total C^5^	**C.**		107.98	77.02	205.43	85.82	75.09	41.62	30.58	24.39
	**P**									
Fructose	**R**		0.36	0.08	0.29	0.06	0.34	0.1	0.42	0.09
Glucose	**O**		0.37	0.1	0.29	0.06	0.35	0.11	0.43	0.1
Sucrose	**P**		0.27	0.18	0.42	0.13	0.31	0.21	0.15	0.18
**Carbohydrate and Amino Acid Ratios**	**R**									
	**A**									
mean C:AA^8^	**T**		50.96	22.97	89.41	62.48	45.55	28.95	42.03	37.72
mean C:EAA^9^	**I**		61.64	47.66	100	89.38	89.37	145.87	103.17	137.14
mean C:nEAA^10^	**O**		422.54	761.96	286.99	282.55	732.98	1882.35	726.34	1120.32

^1^ Plot number and plant species richness level of the sown plant community including *Knautia arvensis*. ^2^ For each plant species the minimum sampling number was five samples per plant species for both analyses, N gives the number of analyzed samples for amino acids and carbohydrates, respectively. ^3^ Volume nectar gives the mean value per inflorescence in µL ± SD (standard deviation). ^4^ Abbreviations: Ala—alanine, Arg—arginine, Asp—aspartic acid, cystine, Glu—glutamic acid, Gly—glycine, His—histidine, Ile—isoleucine, Leu—leucine, Lys—lysine, Met—methionine, Phe—phenylalanine, Pro—proline, Ser—serine, Thr—threonine, Tyr—tyrosine, Val—valine. Order of displayed amino acids reflects the order of appearance in the chromatogram. ^5^ Total AA (amino acids) are the mean sum of all single amino acids ± SD (standard deviation) in mg per mL, followed by individual amino acids. Total C (carbohydrates) are the mean sum of the three main carbohydrates (fructose, glucose, sucrose) in mg/mL ± SD (standard deviation). ^6^ EAA: essential amino acids (His, Thr, Arg, Val, Met, Phe, Ile, Leu, Lys). ^7^ nEAA: non-essential amino acids (Asp, Glu, Ser, Gly, Ala, Tyr, cysteine measured as cystine, Pro). ^8^ Ratio C:AA: ratio of total carbohydrates to total amino acids. ^9^ Ratio C:EAA: ratio of total carbohydrates to essential amino acids. ^10^ Ratio C:nEAA: ratio of total carbohydrates to non-essential amino acids.

**Table 2:** Shannon diversity index [62], total numbers, species richness of flower visitors, number of individuals per flower-visiting guild of *K. arvensis* growing in plant species communities of eight and 16 plant species.

Plant Species Community	8 (B3A20)	8 (B2A12)	16 (B1A20)
Shannon Index	0.72	0.97	0.96
Total Numbers	232	300	383
Species Richness of Flower Visitors	9	12	15
Beetles	-	3	9
Bumblebees	28	49	36
Butterflies	-	2	10
Flies	-	5	2
Honeybees	195	230	303
Hoverflies	6	8	17
Solitary bees	3	2	6
Wasps	-	1	-

## Supplementary Materials

The following are available online at https://www.mdpi.com/2075-4450/11/2/75/s1. Figure S1: Map displaying sizes and locations of plots sampled in this experiment (modified after [48]). Table S1: List of plant species communities with one, four, eight, and 16 plant species, providing plot IDs, sampling year, and sucrose concentration (%) as measured for one sample with a hand-held refractometer, and the numbers and actual species of other plant species flowering and not flowering when *Knautia arvensis* was flowering in 2011. Table S2: List of individual flower visitors to all *Knautia arvensis* in plant species communities with eight and 16 plant species observed in 2011. Figure S2: List of figures presenting the mean (±SD) concentrations and proportions of individual amino acids and carbohydrates as well as total amino acids (AA), all essential amino acids (EAA), and all non-essential amino acids (nEAA) as found in floral nectar of *Knautia arvensis* from different plant species mixtures (i.e., monoculture, four, eight, and 16 plant species mixtures).
