# Supplementary material for: Inter-Individual Nectar Chemistry Changes of Field Scabious, Knautia arvensis
Source: Insects. 2020 Jan 22;11(2):75. doi: 10.3390/insects11020075 (PMC7073839; doi:10.3390/insects11020075)
Supplement: Supplementary file 1 [file insects-11-00075-s001.pdf]

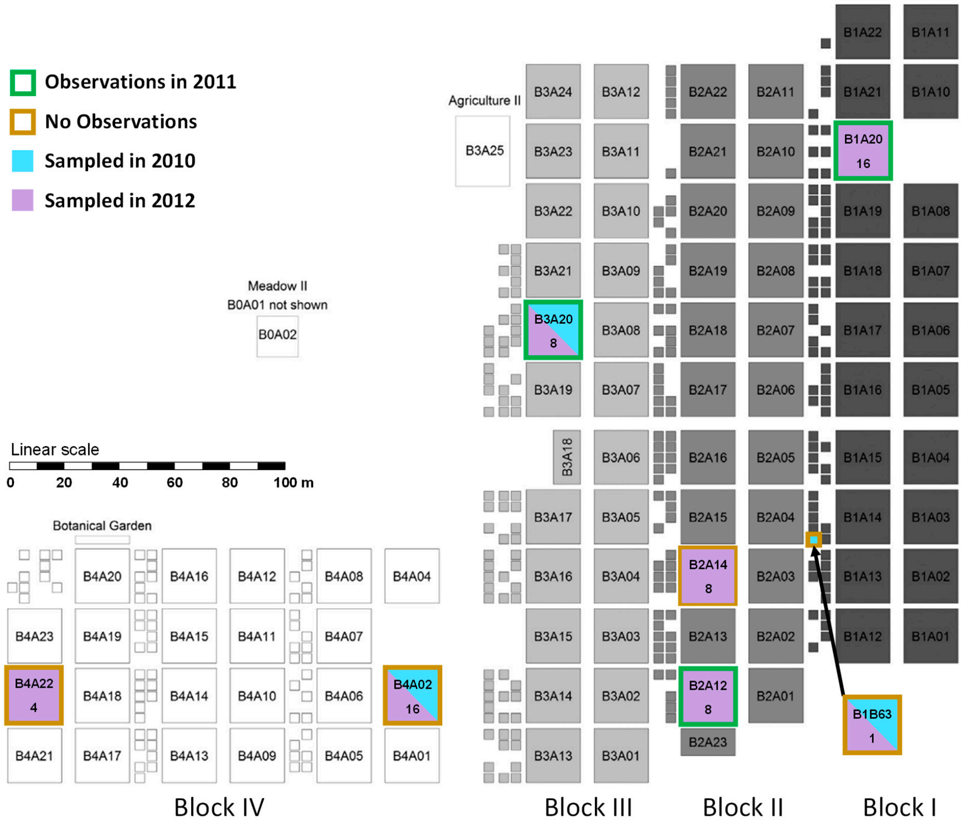

Figure S1. Map displaying sizes and locations of plots sampled in this experiment (modified after [1]). Plot are distributed in four blocks which are perpendicular to the river Saale (to the right). Plots used for pollinator observations in 2011 are indicated by a green frame, no observations by a brown frame. Year of nectar sampling is depicted by colour of the plot filling (blue indicate plots sampled in 2010, violet plots in 2012); note that three plots (B3A20, B1B63, B4A02) were sampled in 2010 and 2012. Numbers below plot IDs in sampled/marked plots give the number of plant species grown in community.

Table S1. List of plant species communities with one, four, eight, and 16 plant species, providing plot IDs, sampling year, sucrose concentration [%] as measured for one sample with a hand-held refractometer, and the numbers and actual species of other plant species flowering and not flowering when *Knautia arvensis* was flowering in 2011. Nectar sampling was performed in 2010 and /or 2012. n.a. indicates that there was no data available.

Plant species mixtures	Plot ID	Year of samp-ling	Sucrose [%]	Number of flowering plant species competing with *K. arvensis*	Flowering plant species	Not flowering plant species
1				0	-	-
	B1B063	2010	27			
		2012	32			
4				2	*Campanula patula*	*Cardamine pratensis*
	B4A22	2012	70			
					Geranium pratense	
8				5	*Anthriscus sylvestris*	*Heracleum sphondylium*
	B2A12	2012	60			
					Galium mollugo	*Sanguisorba officinalis*
					Geranium pratense	
					Leucanthemum vulgare	
					Ranunculus acris	
8				2	*Leontodon hispidus*	*Luzula campestris*
	B2A14	2012	41			
					Veronica chamaedrys	*Phleum pratense*
						Sanguisorba officinalis
						Trifolium dubium
						Trifolium hybridum
8				4	*Heracleum sphondylium*	*Campanula patula*
	B3A20	2010	n. a.			
					Lotus corniculatus	*Cardamine pratensis*
		2012	71			
					Trifolium fragiferum	*Trifolium campestre*
					Trifolium hybridum	
16				10	*Achillea millefolium*	*Ajuga reptans*
	B1A20	2012	57			
					Bellis perennis	*Leontodon autumnalis*
					Geranium pratense	*Medicago varia*
					Leontodon hispidus	*Sanguisorba officinalis*
					Lotus corniculatus	*Trifolium hybridum*
					Onobrychis viciifolia	
					Plantago lanceolata	
					Ranunculus acris	
					Trifolium repens	
					Veronica chamaedrys	
16				5	*Galium mollugo*	*Anthriscus sylvestris*
	B4A02	2010	30			
		2012	30		*Heracleum sphondylium*	*Arrhenatherum elatius*
					Leontodon hispidus	*Cynosurus cristatus*
					Pastinaca sativa	*Glechoma hederacea*

Plantago media	
	Luzula campestris
	Phleum pratense
	Poa pratensis
	Ranunculus acris
	Ranunculus repens
	Taraxacum officinale

Observations of flower visitors (for Table S2 and Table S3)

We related plant species richness to the diversity and abundance of specific flower visitor guilds and the entire flower-visiting community of *Knautia arvensis* (L.) Coult.(Asteraceae) growing in different plant communities, because *K. arvensis* typically attracts many different flower visitors, including solitary bees, bumblebees, honeybees (*Apis mellifera* Linnaeus, 1758) and hoverflies [2–4].

Flower-visiting insects, e.g. honeybees, bumblebees, solitary bees and hoverflies, had been surveyed within the framework of the Jena Experiment on a subset of plots between May and August 2011 (see [5] for details on flower-visitor observations). Thus, nectar sampling and flower-visitor observations were performed at different years. We extracted all observations on flower visitors to *K. arvensis* for those plots for which we also had collected nectar, i.e. two 8-species plots and one 16–species plot. Flower visitors were grouped to honeybees, bumblebees, solitary bees and hoverflies for subsequent analyses, and we defined all solitary bees as non-eusocial Apidae [6]. We summed all flower visitors across all observations performed in 2011 and calculated per plot overall abundance and abundances for different groups as well as overall visitor species richness and the visitor species richness in each group, resulting in a sample size of three.

Notably, the observed and presented visitation patterns should not be considered representative for *Knautia arvensis* in general, as they only provide a snapshot for a largely artificial set-up and for only one year. This is also why this data is only provided as supplementary material.

Table S2. List of individual flower visitors to all *Knautia arvensis* in plant species communities with eight and 16 plant species observed in 2011 (one plot per community with plot number in brackets for further details see [5]. Flower visitors are grouped into beetles, bumblebees, butterflies, flies, honeybees, hoverflies, solitary bees, and wasps (bold letters, alphabetical order). Note that nectar sampling was performed on the same plots, but in different years (2010 and 2012). Observations were performed on the same plots in 2011 from which nectar were taken in 2010 and 2012 for eight and 16 plant species communities.

**Flower visitors of *Knautia arvensis* per species community**	**8 (B3A20)**	**8 (B2A12)**	**16 (B1A20)**
**Beetles**	-	-	-
Coleoptera unidentified	-	3	9
**Bumblebees**			
Bombus lapidarius (Linnaeus, 1758)	14	39	31
Bombus pascuorum (Scopoli, 1763)	-	1	1
Bombus pratorum (Linnaeus, 1761)	8	-	1
Bombus soroeensis (Fabricius, 1776)	-	2	-
Bombus sylvarum (Linnaeus, 1761)	1	-	-
Bombus terrestris (Linnaeus, 1758)	5	4	2
Bombus veteranus (Fabricius, 1793)	-	-	1
Bombus spec.	-	3	-
**Butterflies**			
Araschnia levana (Linnaeus, 1758)	-	-	2
Pieris spec.	-	1	1
Lepidoptera unidentified	-	1	7
**Flies**			
Bombylius venosus (Mikan, 1796)	-	3	-
Brachycera unidentified	-	2	2
**Honeybees**			
Apis mellifera (Linnaeus, 1758)	195	230	303
**Hoverflies**			
Episyrphus balteatus (Dujardin, 1842)	-	3	6
Eristalis jugorum (Egger, 1858)	-	-	-
Eristalis tenax (Linnaeus, 1758)	-	2	-
Melanostoma mellinum (Linnaeus, 1758)	1	-	2
Scaeva pyrastri (Linnaeus, 1758)	2	-	-
Syritta pipiens (Linnaeus, 1758)	1	-	-
Syrphidae unidentified	2	3	9
**Solitary bees**			
Andrena cineraria (Linnaeus, 1758)	-	-	-
Andrena hattorfiana (Fabricius, 1775)	-	-	-
Halictus scabiosae (Rossi, 1790)	-	-	1
Lasioglossum calceatum (Scopoli, 1763)	-	-	1
Lasioglossum leucozonium (Schrank, 1781)	-	2	-
Lasioglossum villosulum (Kirby, 1802)	-	-	1
Solitary bees unidentified	3	-	3
**Wasps**			
Lindenius spec.	-	1	-

Table S3. Total numbers, species richness, guild numbers and Shannon diversity [7] of flower visitors to *Knautia arvensis* growing in plant species communities of four, eight, and 16 plant species.

**Plant species community**	**8**	**8**	**16**
**Shannon Index**	**0.72**	**0.97**	**0.96**
**Total numbers**	**232**	**300**	**383**
**Species richness of flower visitors**	**9**	**12**	**15**
Beetles	-	3	9
Bumblebees	28	49	36
Butterflies	-	2	10
Flies	-	5	2
Honeybees	195	230	303
Hoverflies	6	8	17
Solitary bees	3	2	6
**Wasps**	-	1	-

Commented [AK1]: Ist das nicht eher "number of individuals per flower-visiting guild"?

Unter "guild numbers" würde ich die Anzahl der verschiedenen Guilden bezeichnen und dies wäre falsch

Commented [AK2]: Warum sind manche in bold? Dies müsste hier auch noch stehen.

Commented [AK3]: Muss hier nicht eine 4 stehen?

Figure S2. List of figures presenting the mean [± SD] concentrations and proportions of individual amino acids and carbohydrates as well as total amino acids (AA), all essential amino acids (EAA) and all non-essential amino acids (nEAA) as found in floral nectar of *Knautia arvensis* from different plant species mixtures, i.e. monoculture, four, eight, and 16 plant species mixtures. The figure correspond to Table 1.

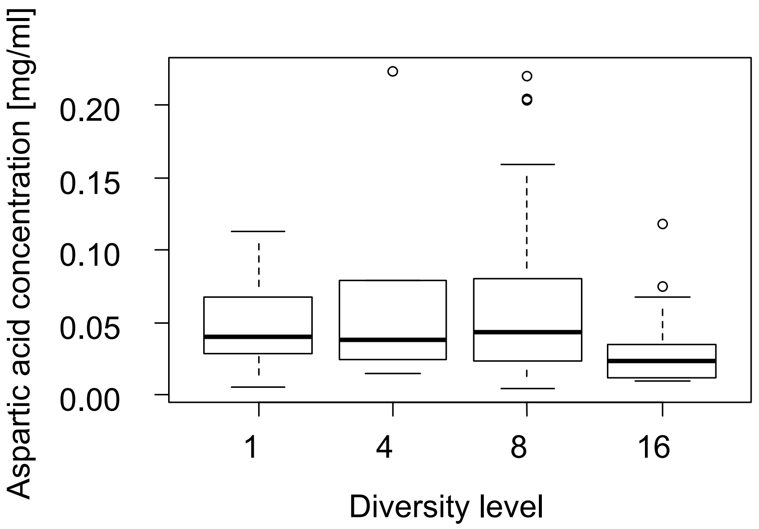

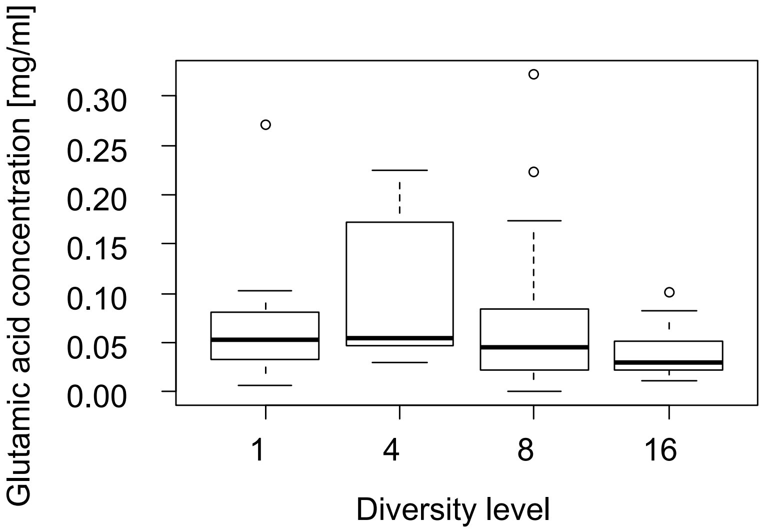

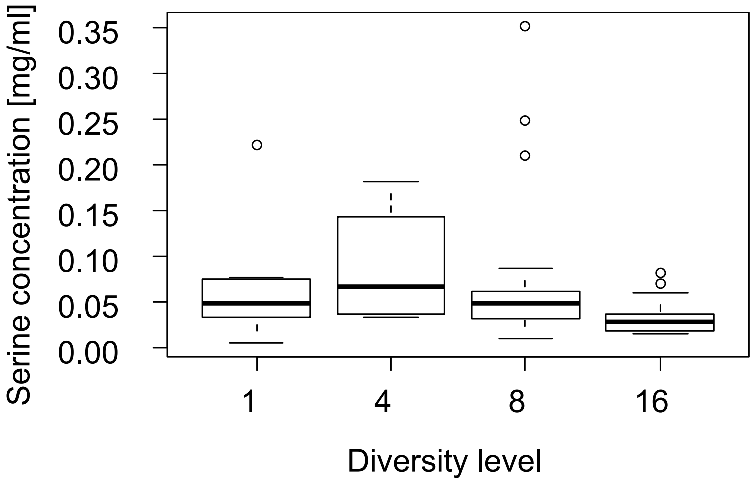

Serine concentration [mg/ml]
0.35
0.30
0.25
0.20
0.15
0.10
0.05
0.00
1
4
8
16
Diversity level

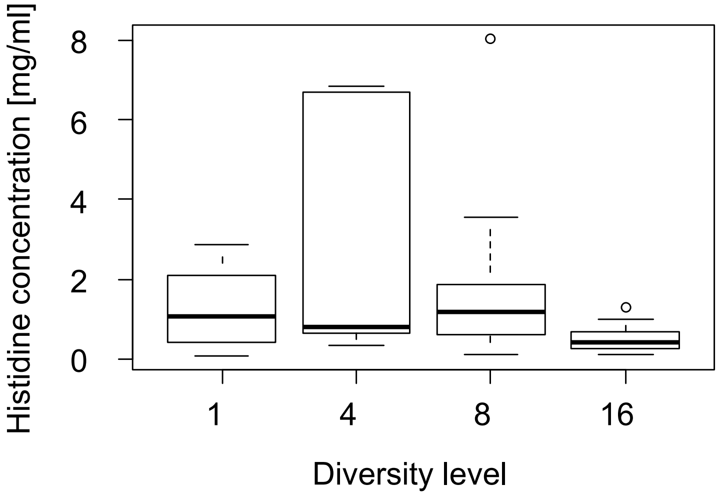

Histidine concentration [mg/ml]
8
6
4
2
0
1
4
8
16
Diversity level

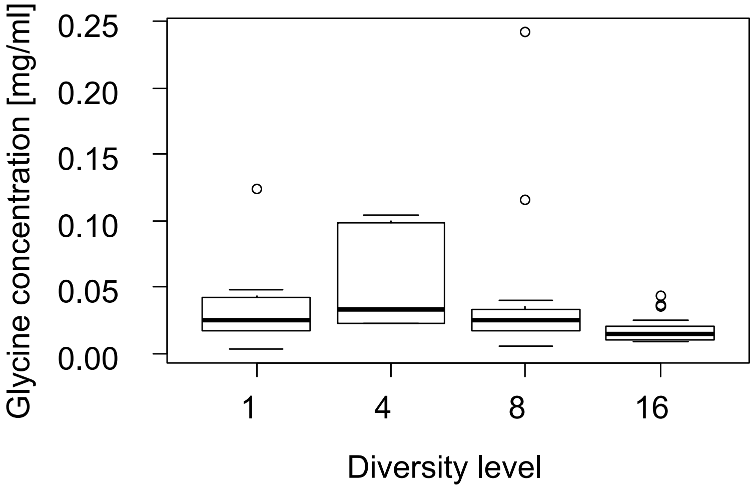

Glycine concentration [mg/ml]
0.25
0.20
0.15
0.10
0.05
0.00
1
4
8
16
Diversity level

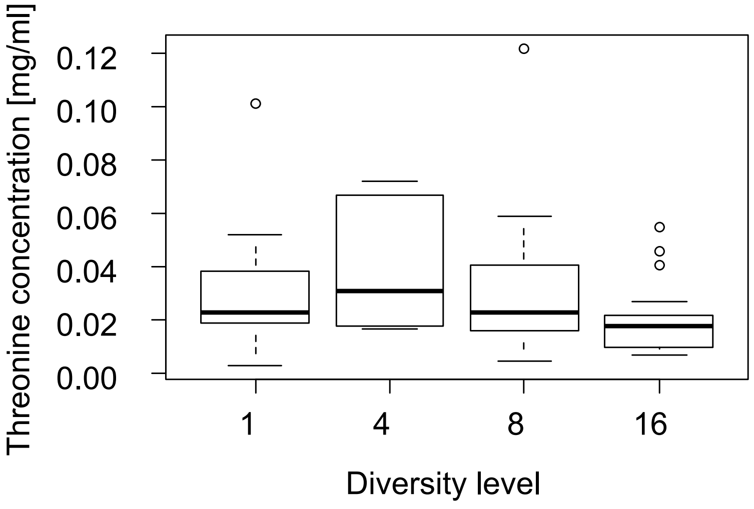

Threonine concentration [mg/ml]
0.12
0.10
0.08
0.06
0.04
0.02
0.00
1
4
8
16
Diversity level

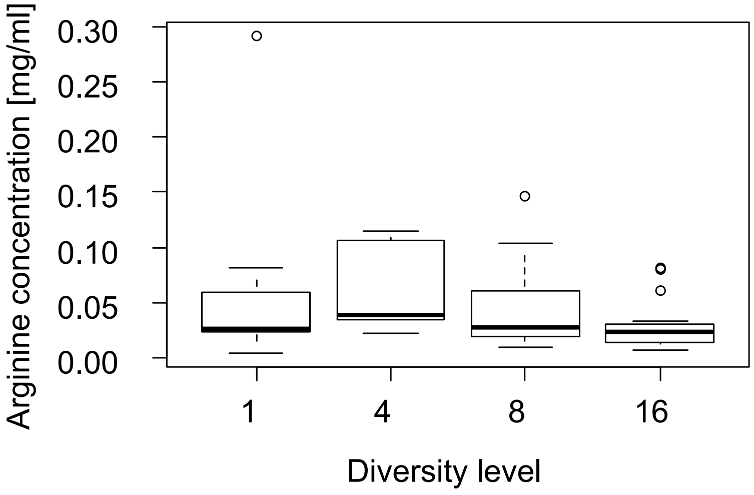
Arginine concentration [mg/ml]
0.30
0.25
0.20
0.15
0.10
0.05
0.00
1
4
8
16
Diversity level

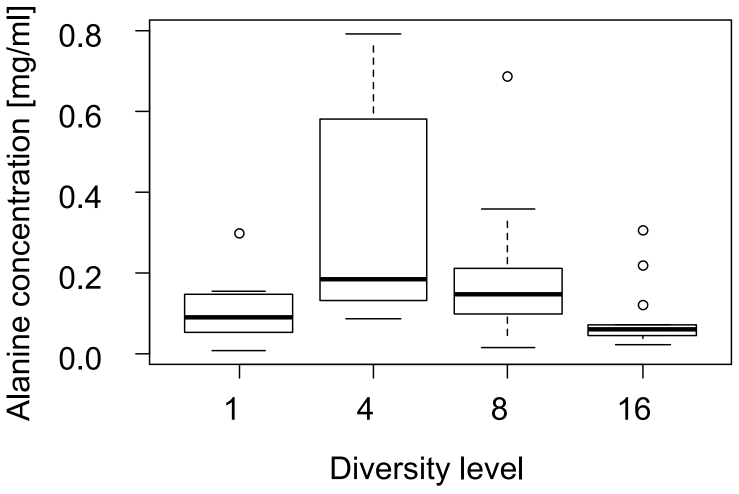
Alanine concentration [mg/ml]
0.8
0.6
0.4
0.2
0.0
1
4
8
16
Diversity level

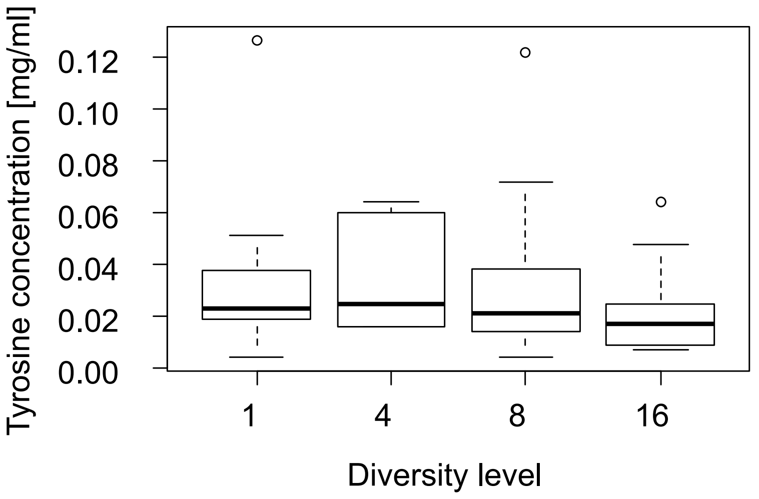

Tyrosine concentration [mg/ml]
0.12
0.10
0.08
0.06
0.04
0.02
0.00
1
4
8
16
Diversity level

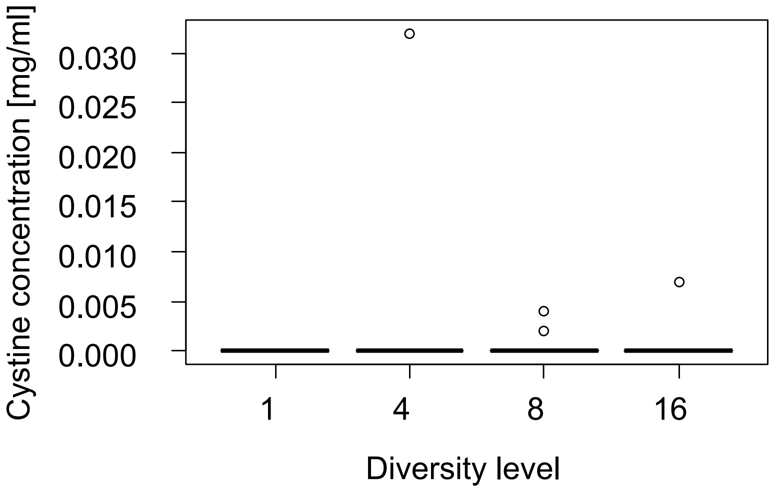

Cystine concentration [mg/ml]
0.030
0.025
0.020
0.015
0.010
0.005
0.000
1
4
8
16
Diversity level

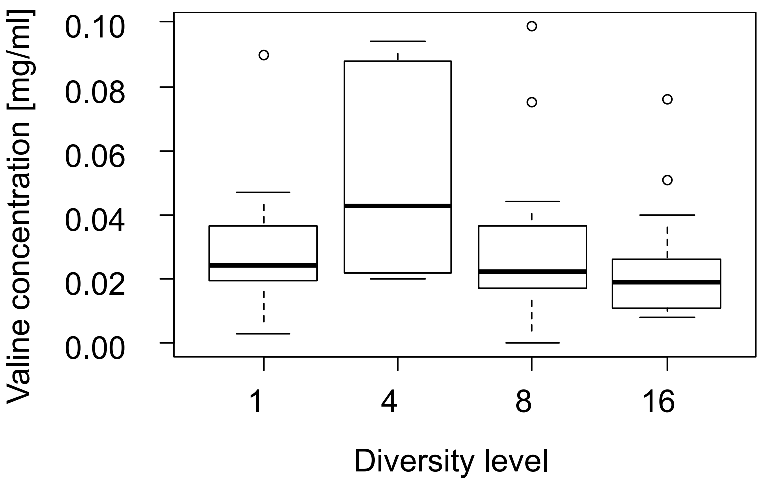
Valine concentration [mg/ml]
0.10
0.08
0.06
0.04
0.02
0.00
1
4
8
16
Diversity level

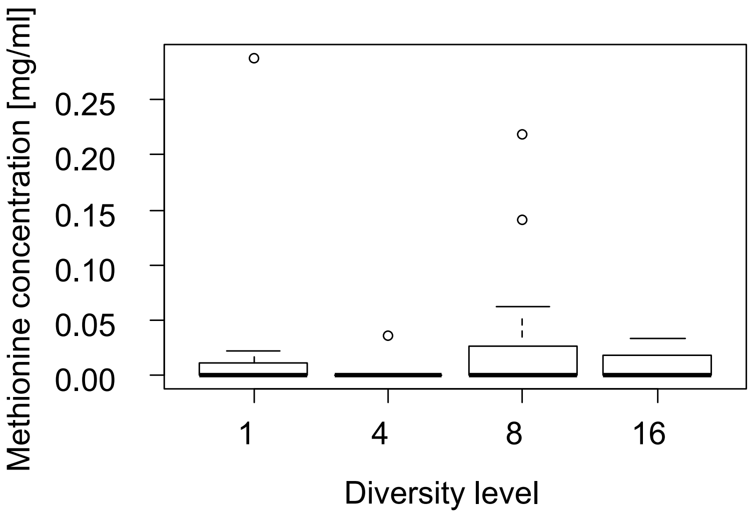
Methionine concentration [mg/ml]
0.25
0.20
0.15
0.10
0.05
0.00
1
4
8
16
Diversity level

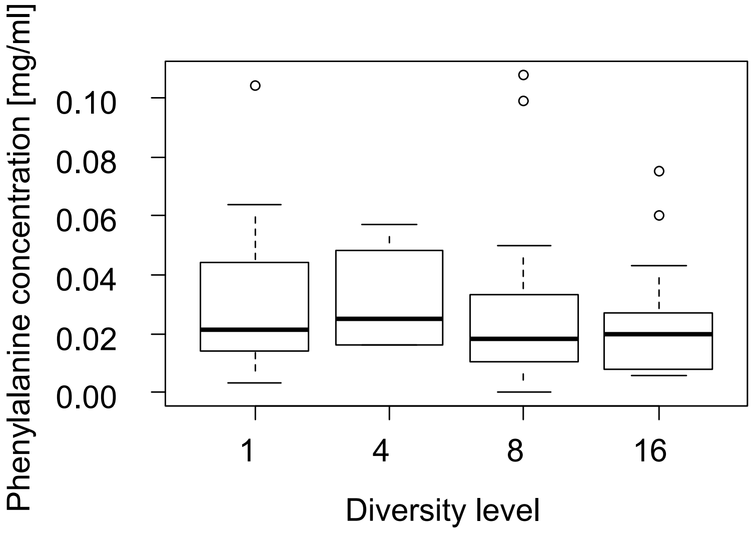
Phenylalanine concentration [mg/ml]
0.00
0.02
0.04
0.06
0.08
0.10
1
4
8
16
Diversity level

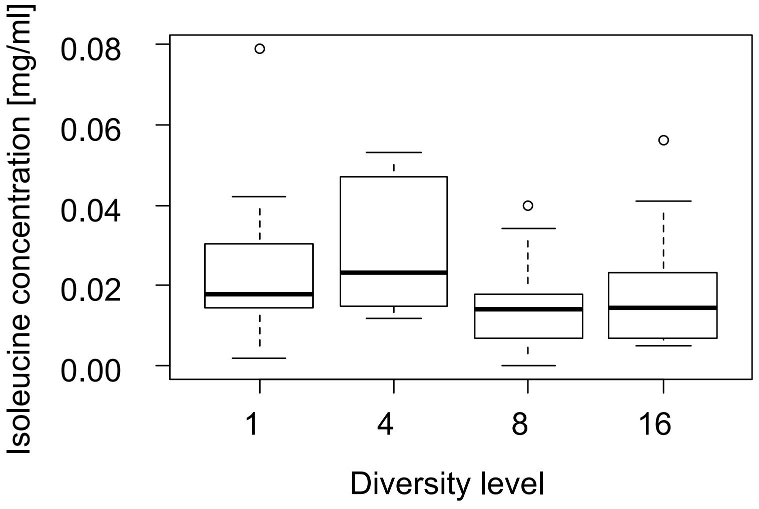
Isoleucine concentration [mg/ml]
0.00
0.02
0.04
0.06
0.08
1
4
8
16
Diversity level

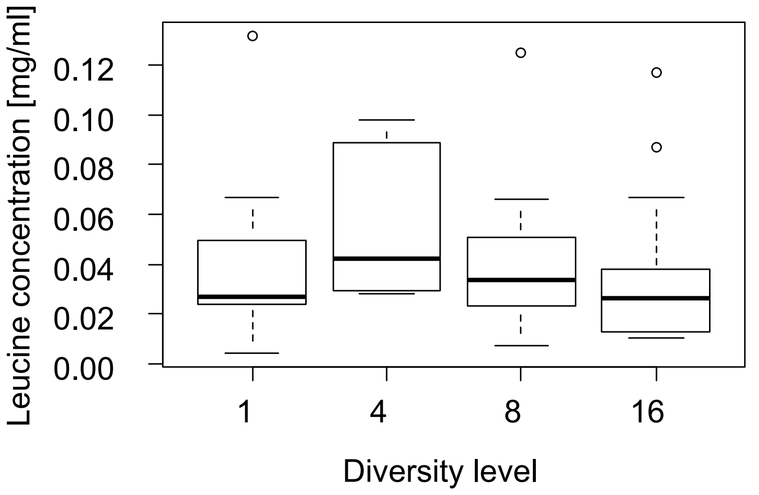

Leucine concentration [mg/ml]
0.00
0.02
0.04
0.06
0.08
0.10
0.12
1
4
8
16
Diversity level

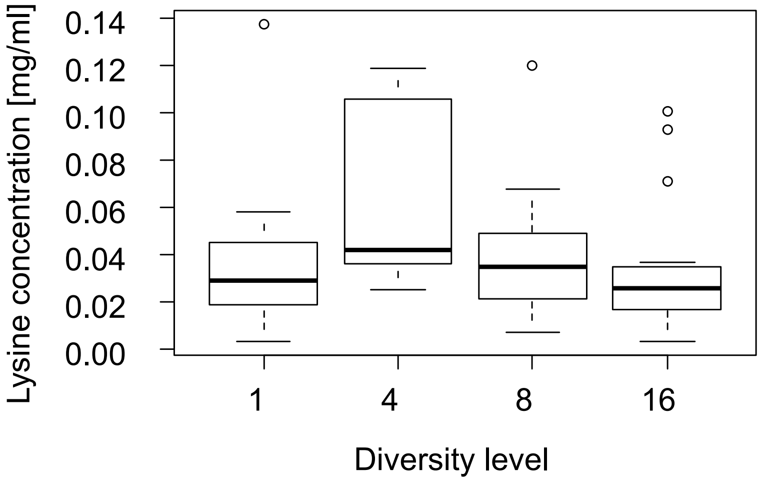

Lysine concentration [mg/ml]
0.00
0.02
0.04
0.06
0.08
0.10
0.12
0.14
1
4
8
16
Diversity level

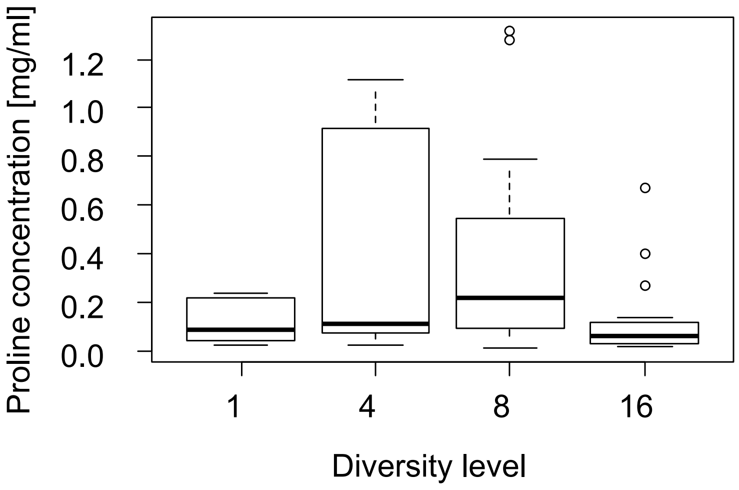

Proline concentration [mg/ml]
1.2
1.0
0.8
0.6
0.4
0.2
0.0
1
4
8
16
Diversity level

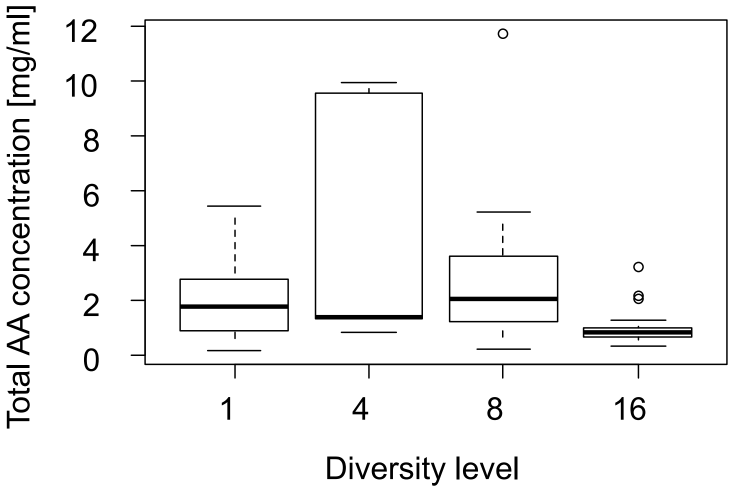

Total AA concentration [mg/ml]
12
10
8
6
4
2
0
1
4
8
16
Diversity level

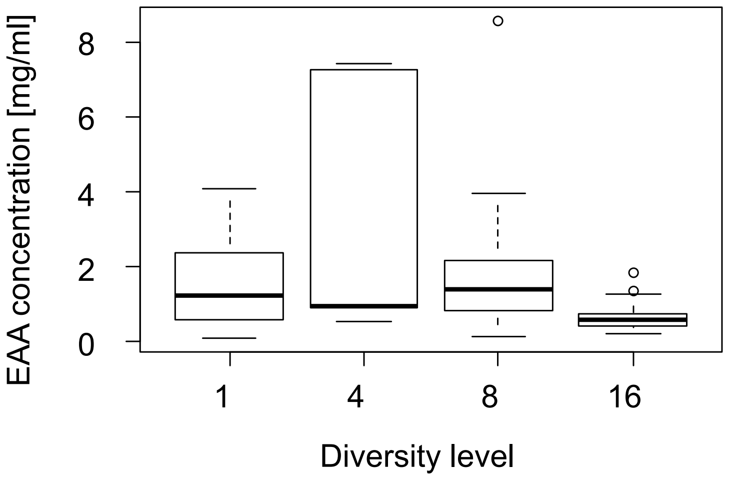
EAA concentration [mg/ml]
0
2
4
6
8
1
4
8
16
Diversity level

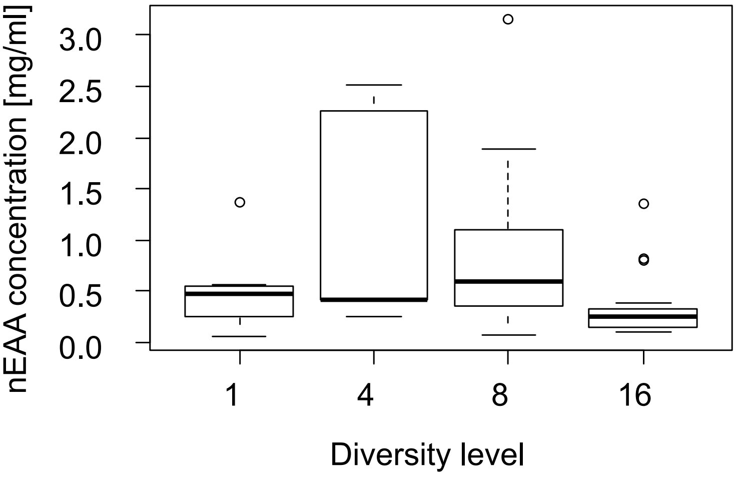
nEAA concentration [mg/ml]
0.0
0.5
1.0
1.5
2.0
2.5
3.0
1
4
8
16
Diversity level

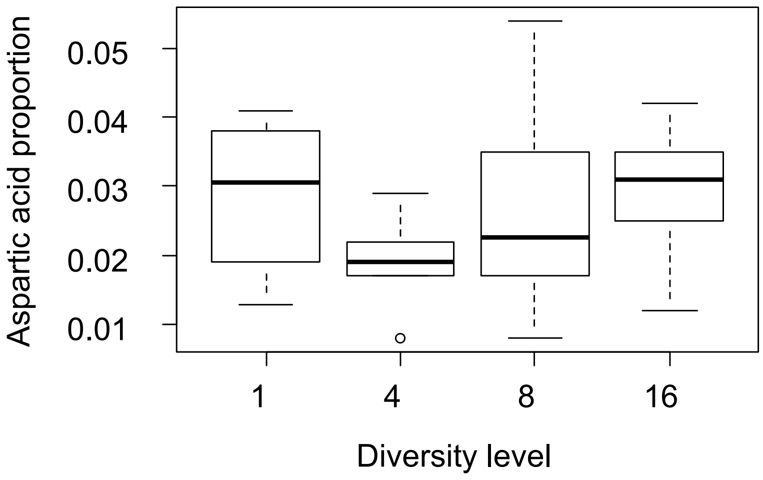
Aspartic acid proportion
0.05
0.04
0.03
0.02
0.01
1
4
8
16
Diversity level

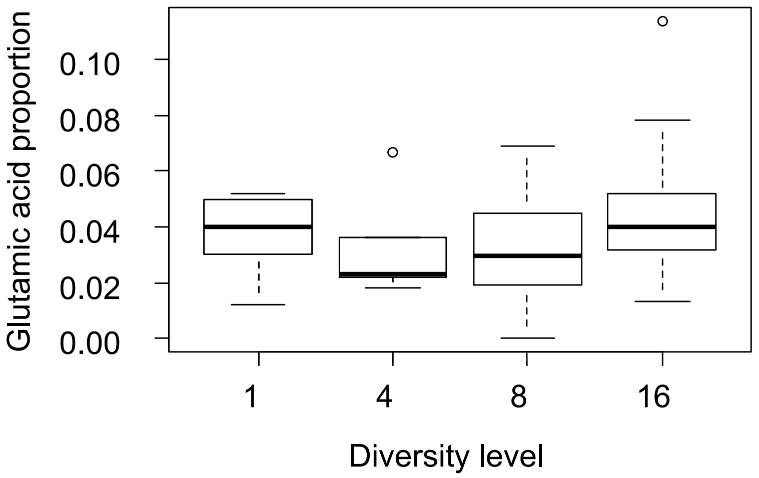
Glutamic acid proportion
0.10
0.08
0.06
0.04
0.02
0.00
1
4
8
16
Diversity level

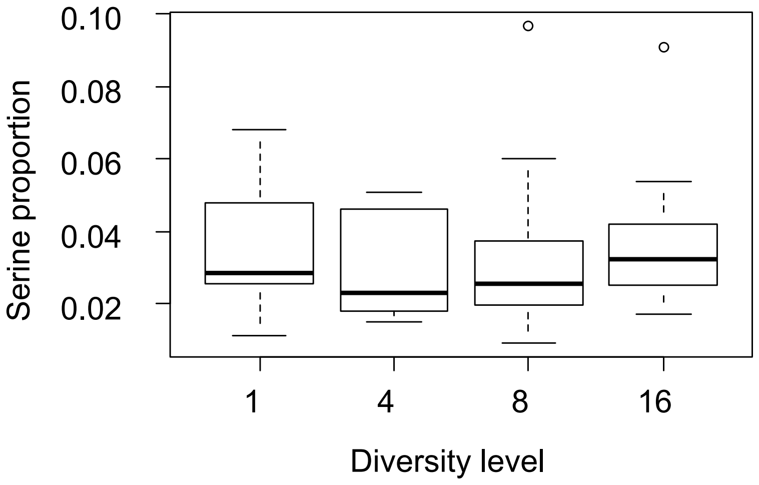

0.10
0.08
0.06
0.04
0.02
Serine proportion
1
4
8
16
Diversity level

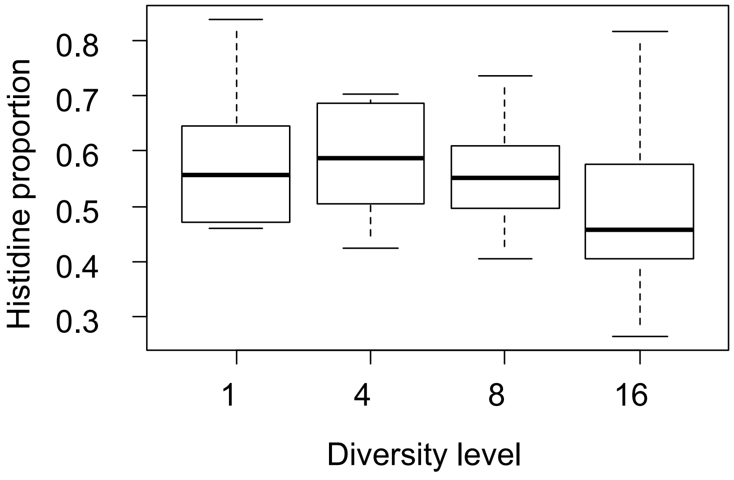

0.8
0.7
0.6
0.5
0.4
0.3
Histidine proportion
1
4
8
16
Diversity level

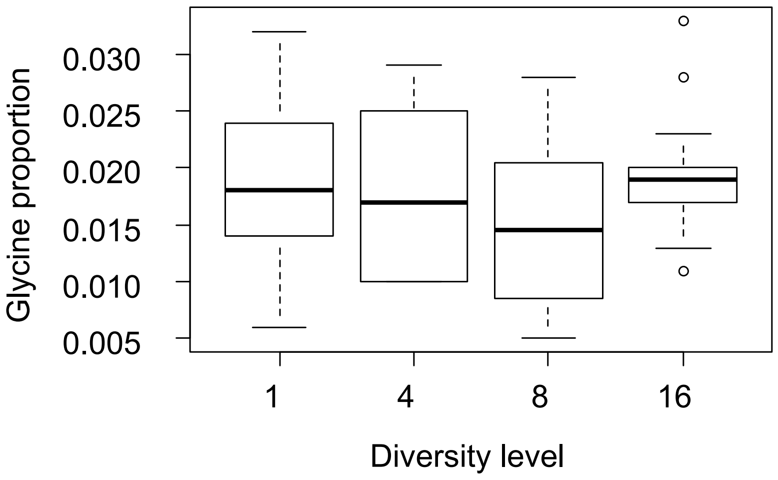
Glycine proportion
0.005
0.010
0.015
0.020
0.025
0.030
1
4
8
16
Diversity level

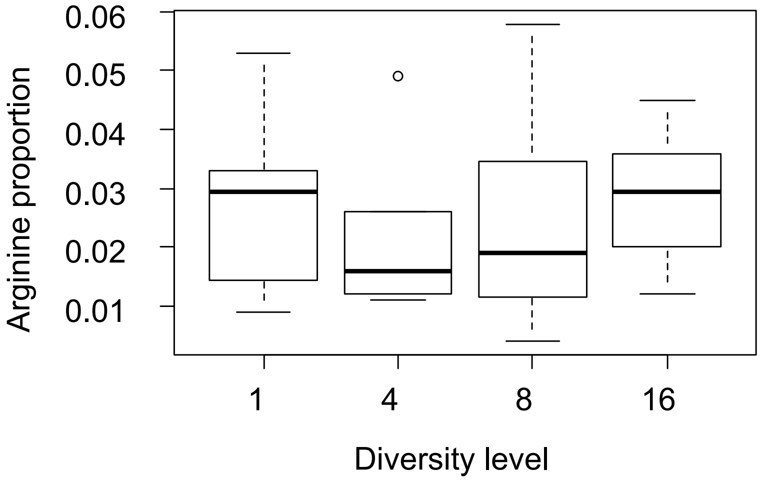
Arginine proportion
0.01
0.02
0.03
0.04
0.05
0.06
1
4
8
16
Diversity level

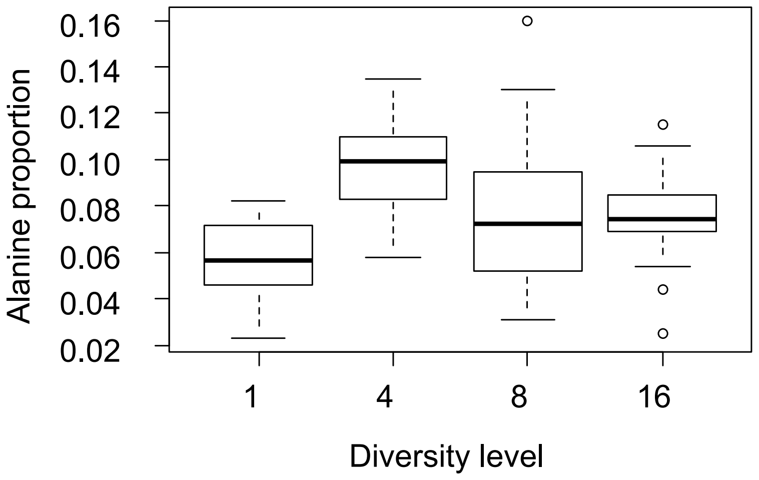
0.16
0.14
0.12
0.10
0.08
0.06
0.04
0.02
Alanine proportion
1
4
8
16
Diversity level

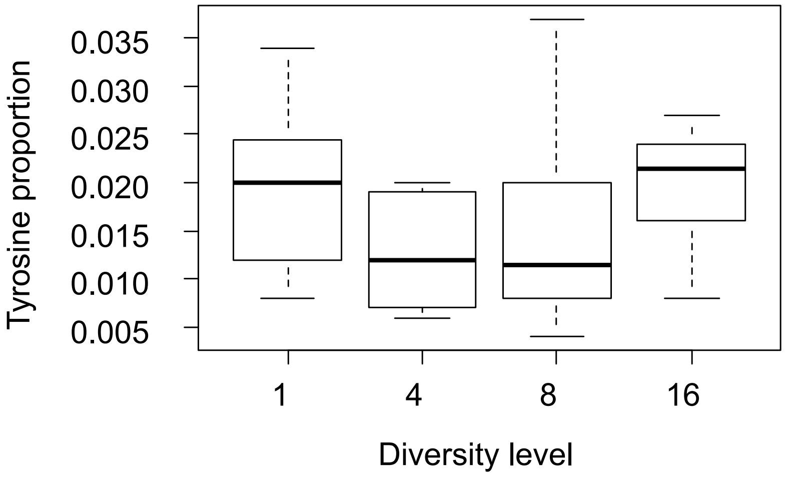
0.035
0.030
0.025
0.020
0.015
0.010
0.005
Tyrosine proportion
1
4
8
16
Diversity level

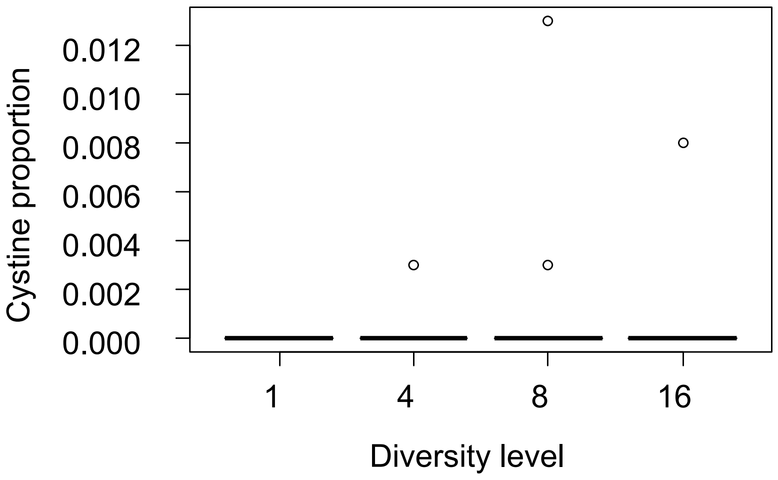
0.012
0.010
0.008
0.006
0.004
0.002
0.000
Cystine proportion
1
4
8
16
Diversity level

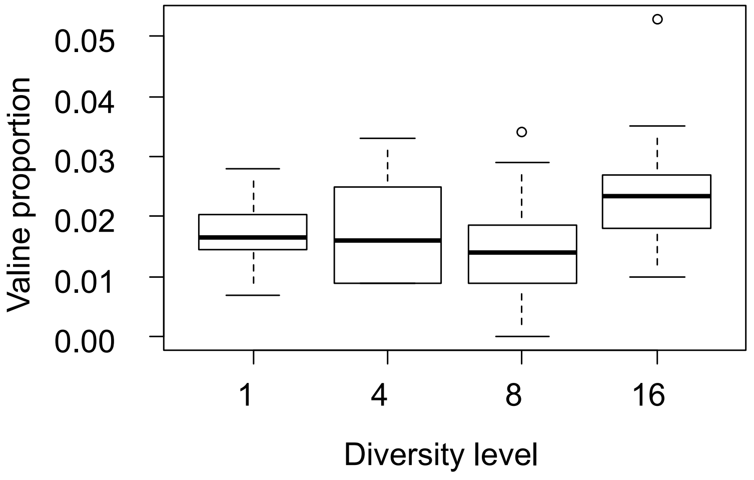
0.05
0.04
0.03
0.02
0.01
0.00
Valine proportion
1
4
8
16
Diversity level

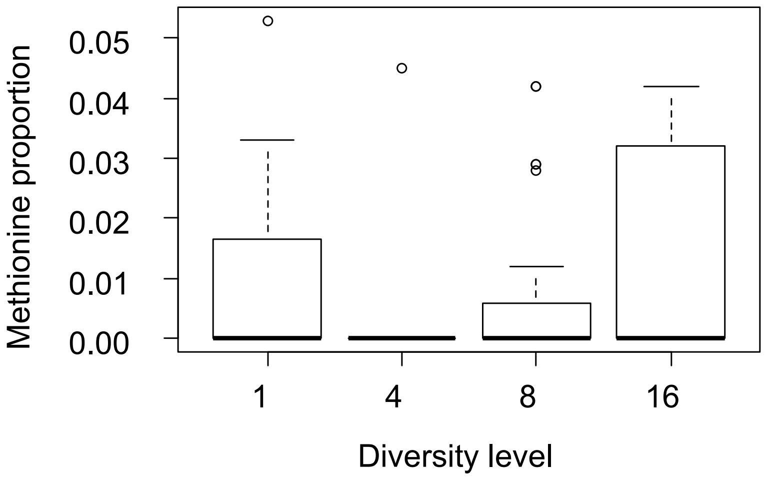

Methionine proportion
0.00
0.01
0.02
0.03
0.04
0.05
1
4
8
16
Diversity level

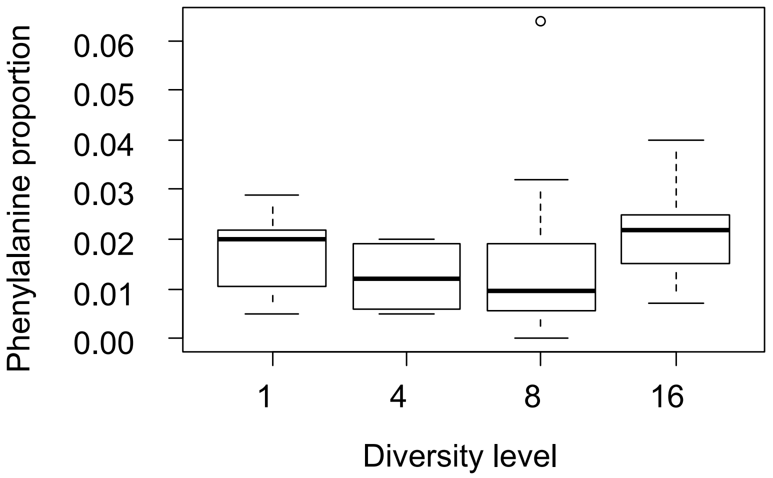

Phenylalanine proportion
0.00
0.01
0.02
0.03
0.04
0.05
0.06
1
4
8
16
Diversity level

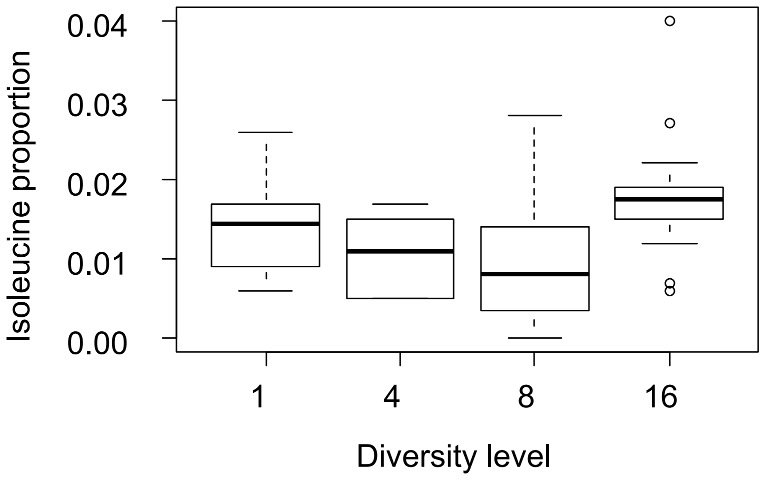
0.04
0.03
0.02
0.01
0.00
Isoleucine proportion
1
4
8
16
Diversity level

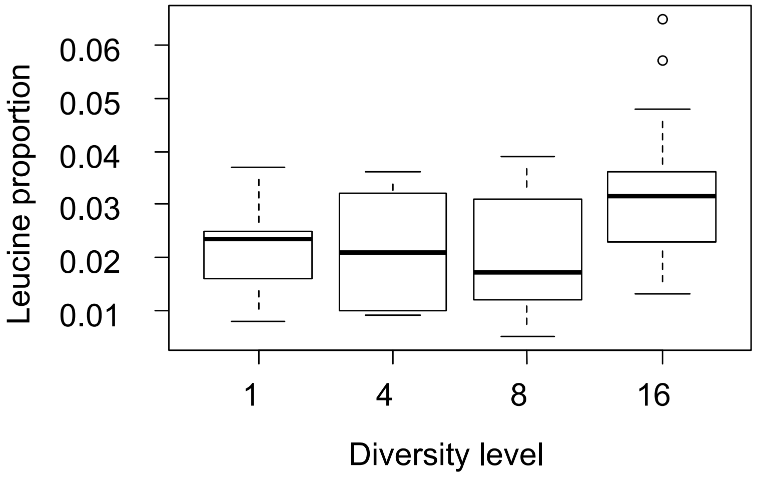
0.06
0.05
0.04
0.03
0.02
0.01
Leucine proportion
1
4
8
16
Diversity level

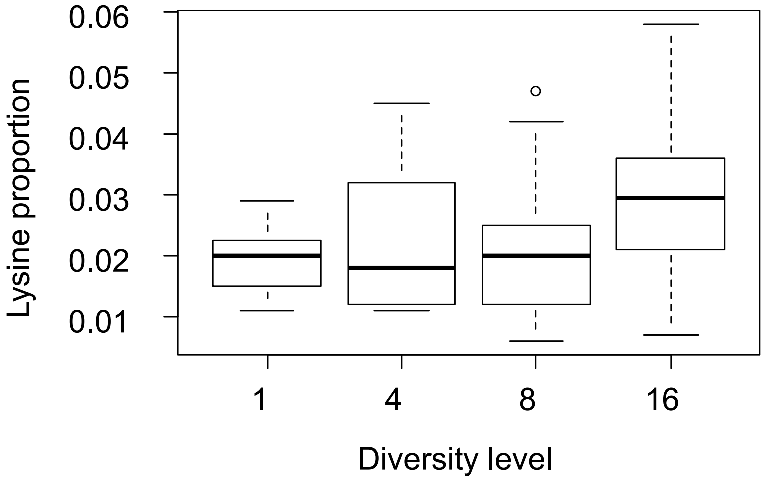

0.06
0.05
0.04
0.03
0.02
0.01
Lysine proportion
1
4
8
16
Diversity level

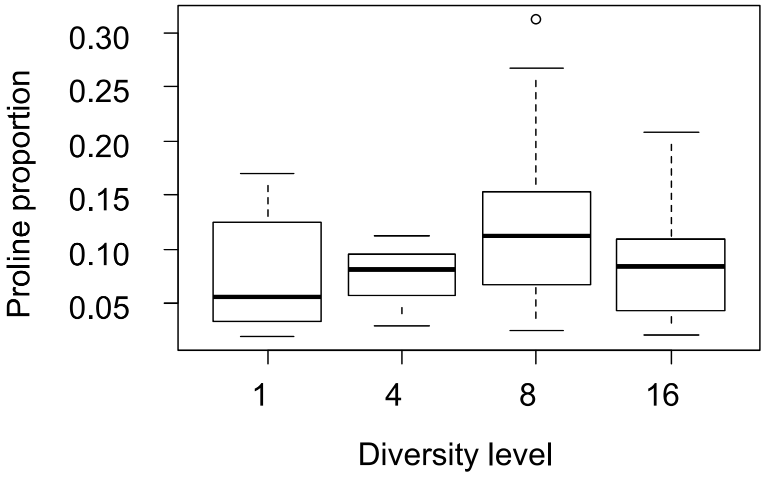

0.30
0.25
0.20
0.15
0.10
0.05
Proline proportion
1
4
8
16
Diversity level

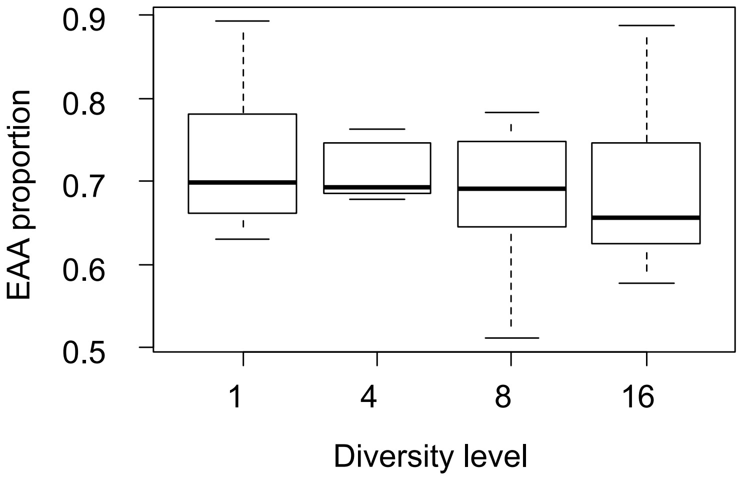
0.9
0.8
0.7
0.6
0.5
EAA proportion
1
4
8
16
Diversity level

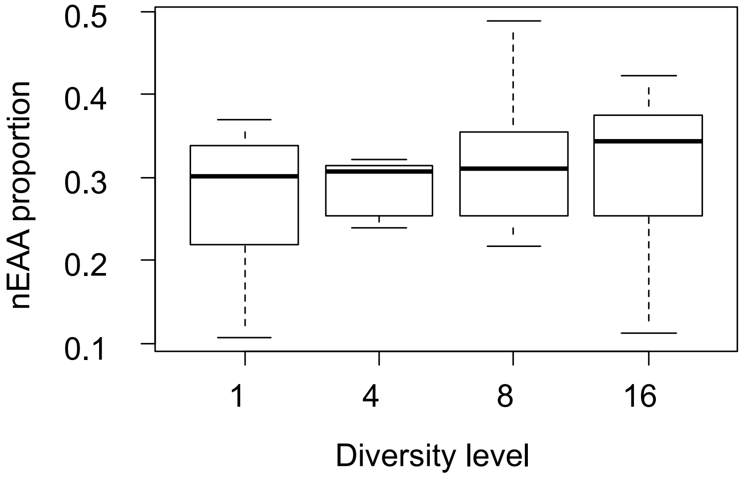
0.5
0.4
0.3
0.2
0.1
nEAA proportion
1
4
8
16
Diversity level

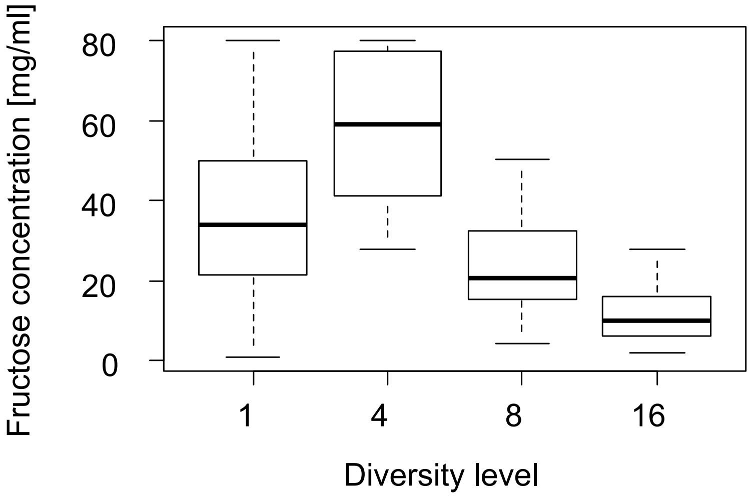
Fructose concentration [mg/ml]
0
20
40
60
80
1
4
8
16
Diversity level

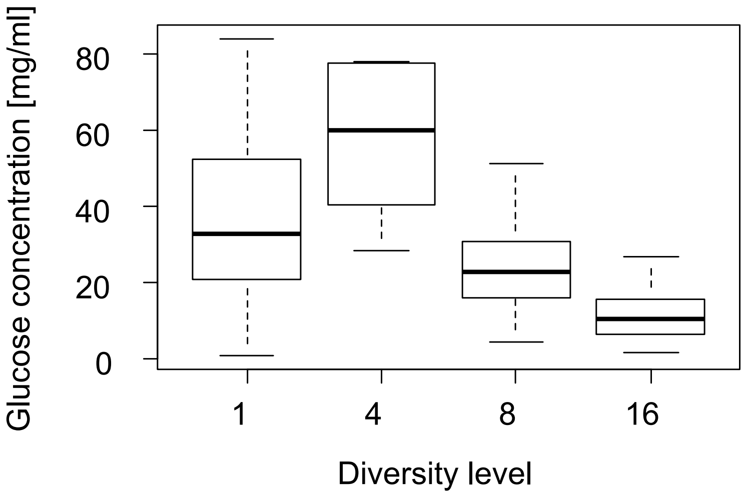
Glucose concentration [mg/ml]
0
20
40
60
80
1
4
8
16
Diversity level

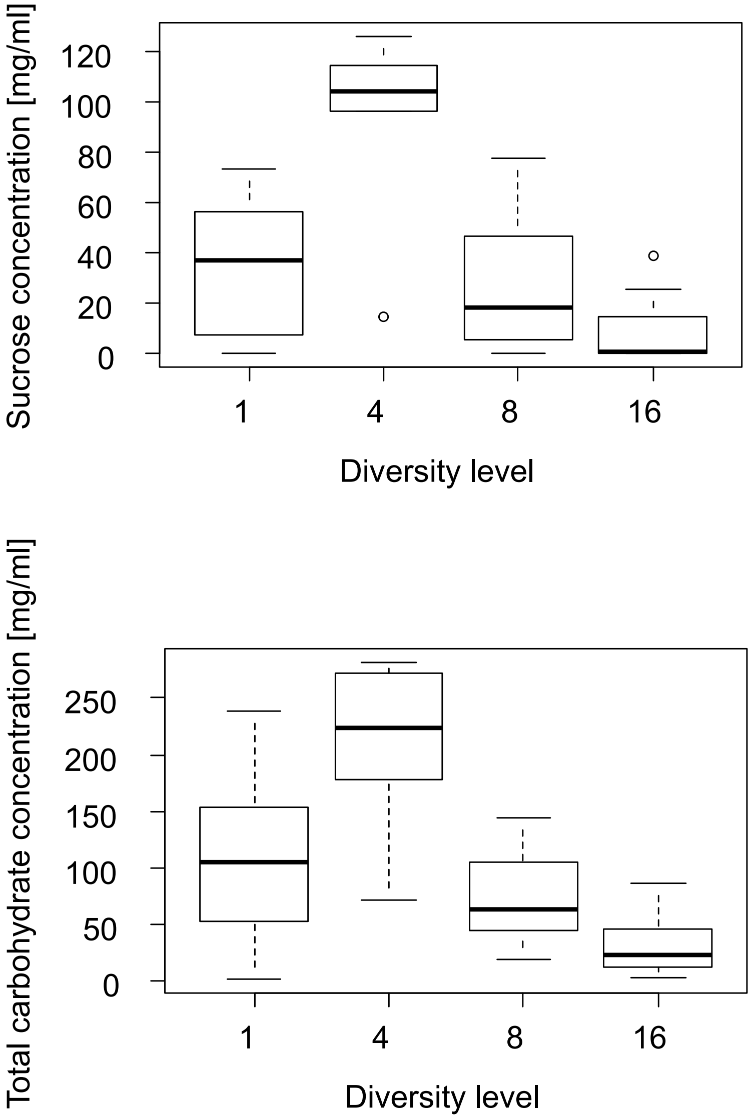
Sucrose concentration [mg/ml]
0
20
40
60
80
100
120
1
4
8
16
Diversity level
Total carbohydrate concentration [mg/ml]
0
50
100
150
200
250
1
4
8
16
Diversity level

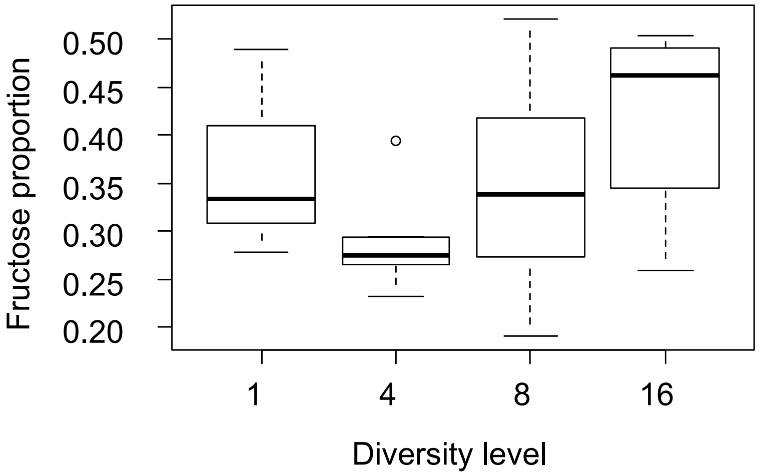
0.50
0.45
0.40
0.35
0.30
0.25
0.20
Fructose proportion
1
4
8
16
Diversity level

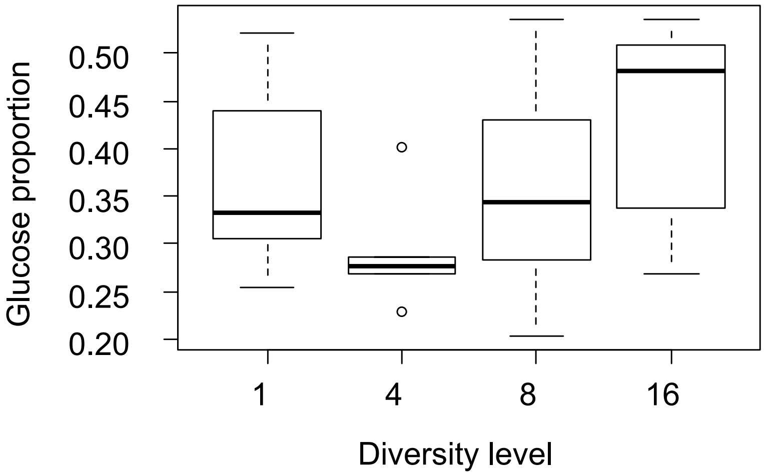
0.50
0.45
0.40
0.35
0.30
0.25
0.20
Glucose proportion
1
4
8
16
Diversity level

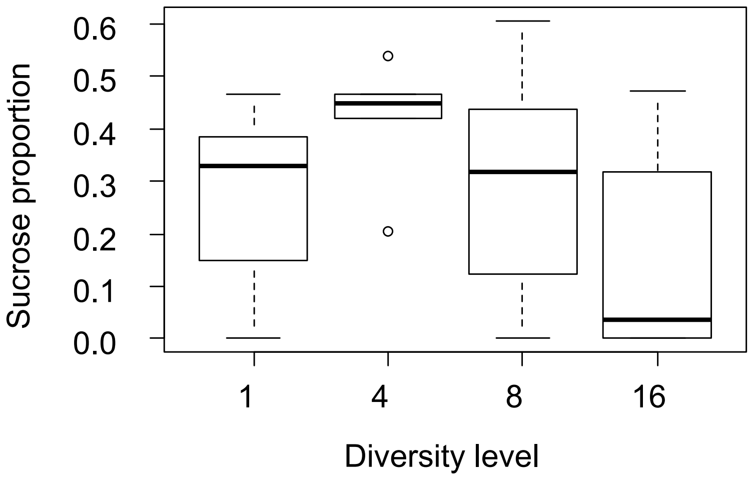
Sucrose proportion
0.0
0.1
0.2
0.3
0.4
0.5
0.6
1
4
8
16
Diversity level

References

1. Weisser, W.W.; Roscher, C.; Meyer, S.T.; Ebeling, A.; Luo, G.; Allan, E.; Beßler, H.; Barnard, R.L.; Buchmann, N.; Buscot, F.; et al. Biodiversity effects on ecosystem functioning in a 15-year grassland experiment: Patterns, mechanisms, and open questions. *Basic Appl. Ecol.* **2017**, *23*, 1–73.

2. Ebeling, A.; Klein, A.-M.; Tscharntke, T. Plant–flower visitor interaction webs: Temporal stability and pollinator specialization increases along an experimental plant diversity gradient. *Basic Appl. Ecol.* **2011**, *12*, 300–309.

3. Larsson, M. Higher pollinator effectiveness by specialist than generalist flower-visitors of unspecialized Knautia arvensis (Dipsacaceae). *Oecologia* **2005**, *146*, 394–403.

4. Knuth, P.E.O.W.; Appel, O.; Loew, E.; Müller, H. *Handbuch der Blütenbiologie Band 2, Teil 1*; Engelmann: Leipzig, 1898.

5. Venjakob, C.; Klein, A.-M.; Ebeling, A.; Tscharntke, T.; Scherber, C. Plant diversity increases spatio-temporal niche complementarity in plant-pollinator interactions. *Ecol. Evol.* **2016**, *6*, 2249–2261.

6. Brittain, C.; Kremen, C.; Klein, A.-M. Biodiversity buffers pollination from changes in environmental conditions. *Glob. Chang. Biol.* **2013**, *19*, 540–7.

7. Magurran, A.E. *Measuring Biological Diversity*; Blackwell Science: Oxford, 2004; ISBN 978-0-632-05633-0.
